# Reimagining plant science training in the era of generative artificial intelligence: a global perspective

**DOI:** 10.1093/plcell/koag140

**Published:** 2026-05-12

**Authors:** Gaurav D Moghe, Alen Zimić-Sheen, Dijun Chen, Gitanjali Yadav, Guangshuo Cao, Hale Tufan, Jason Williams, Jędrzej Szymański, Jeongwoon Kim, Lucas Busta, Marek Mutwil, Miguel Verdú, Mirko Zimić, Nicholas J Provart, Nokwanda Makunga, Olivia Wilkins, Qi Sun, Robert VanBuren, Rose A Marks, Seung Y Rhee, Yu Jiang, Yuying Xie

**Affiliations:** Plant Biology Section, School of Integrative Plant Science, Cornell University, Ithaca, NY, United States; Plant Biology Section, School of Integrative Plant Science, Cornell University, Ithaca, NY, United States; State Key Laboratory of Pharmaceutical Biotechnology, School of Life Sciences, Nanjing University, Nanjing, China; Biodiversity Informatics Laboratory, National Institute of Plant Genome Research, New Delhi, India; State Key Laboratory of Pharmaceutical Biotechnology, School of Life Sciences, Nanjing University, Nanjing, China; Plant Breeding and Genetics Section, School of Integrative Plant Science, Cornell University, Ithaca, NY, United States; DNA Learning Center, Cold Spring Harbor Laboratory, Cold Spring Harbor, NY, United States; Leibniz Institute of Plant Genetics and Crop Plant Research (IPK), Seeland 06466, Germany; Forschungszentrum Jülich, Institute of Bio- and Geosciences (IBG-4 Bioinformatics), CEPLAS, BioSC, Jülich, Germany; Bayer Crop Science, St.Louis, MO, United States; Department of Chemistry and Biochemistry, University of Minnesota—Duluth, Duluth, MN, United States; Department of Plant and Environmental Sciences, University of Copenhagen, Copenhagen, Denmark; Centro de Investigaciones Sobre Desertificación, CSIC-UV-GV, Valencia, Spain; Laboratorio de Bioinformática y Biología Molecular, Facultad de Ciencias e Ingeniería, Universidad Peruana Cayetano Heredia, Lima, Peru; Department of Cell & Systems Biology/Centre for the Analysis of Genome Evolution and Function, University of Toronto, Toronto, Ontario, Canada; Department of Botany and Zoology, Stellenbosch University, Private Bag X1, Matieland 7600, South Africa; Department of Biological Science, University of Manitoba, Winnipeg, Manitoba, Canada; Bioinformatics Facility, Institute of Biotechnology, Cornell University, Ithaca, NY, United States; Department of Plant Biology, Michigan State University, East Lansing, MI, United States; Department of Plant, Soil, and Microbial Sciences, Michigan State University, East Lansing, MI, United States; Plant Resilience Institute, Michigan State University, East Lansing, MI, United States; Department of Plant Biology, University of Illinois Urbana-Champaign, Urbana, IL, United States; Department of Plant Biology, Michigan State University, East Lansing, MI, United States; Department of Plant, Soil, and Microbial Sciences, Michigan State University, East Lansing, MI, United States; Plant Resilience Institute, Michigan State University, East Lansing, MI, United States; Department of Biochemistry and Molecular Biology, Michigan State University, East Lansing, MI, United States; Horticulture Section, School of Integrative Plant Science, Cornell AgriTech, Cornell University, Ithaca, NY, United States; Department of Computational Mathematics, Science, and Engineering, Michigan State University, East Lansing, MI, United States

## Abstract

In recent years, a deluge of big and diverse datasets from hundreds of plant species, coupled with spectacular innovations in artificial intelligence (AI) and generative AI (GenAI), has altered the landscape of plant science. These developments are increasingly democratizing the field, reducing the entry barriers to complex data analysis and enabling a new wave of innovative research while introducing new challenges. Therefore, in this era, it is critical that we train the next generation of plant scientists to be AI-literate, ie, not only proficient in using AI but also vigilant about its pitfalls and biases. In this perspective, we call for six strategic shifts necessary for training the next generation of plant scientists. We argue that while maintaining a core focus on subject expertise, educators should simultaneously emphasize development of new AI-forward pedagogical and evaluation frameworks that reward interdisciplinary and critical thinking, human-driven knowledge synthesis, self-directed learning, and conceptual understanding of workflows. For effective critique and sound interpretations based on biological reality, plant scientists must be explicitly trained in recognizing biases underlying GenAI models. Finally, we highlight the structural barriers hindering the equitable and ethical use of GenAI, where awareness and resolution are critical for sustainable growth of the field. Through the above conceptual framework and numerous plant-science-focused illustrative activities, examples, and resources meant for students and educators alike, this Perspective defines high-level emphasis areas for GenAI-enabled scientific training, aimed at creating a more effective, engaged, and adaptive community of plant scientists.

## Introduction

**Figure 1:**
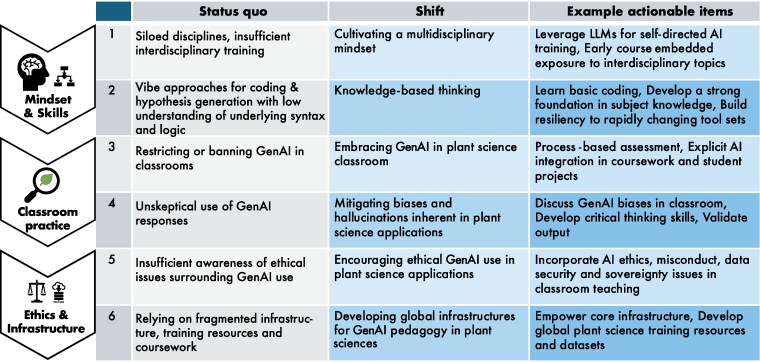
A roadmap to GenAI literacy in plant sciences. Current challenges include siloed disciplines, uncritical vibe-coding, reliance on biased data and insufficient AI knowledge. Six strategic shifts are proposed to transform plant science education and research, moving from passive use to active, knowledge-based, ethical engagement. The outcome is a global, interconnected community where GenAI complements disciplinary knowledge to solve complex plant science problems.

Generative artificial intelligence (GenAI) (Box 1), which includes large language models (LLMs), marks a transformative shift in how we generate knowledge, solve problems, and communicate science. It is also fundamentally altering the landscape of plant science research and education. LLMs are being increasingly used for learning, coursework, hypothesis generation, data analysis, coding, as well as manuscript and grant writing. While GenAI has unparalleled utility in these contexts, its uncritical or malicious use can be dangerous—ranging from a surge of publications with questionable quality, ie, “AI slop” (Down 2025), to accidental generation of nonexistent citations, irrelevant hypotheses, erroneous experimental designs, and incorrect inferences. For educators too, the classroom has become a complex frontier: traditional evaluation methods, such as report writing, quizzes, and coding assignments can often be rendered ineffective by LLMs, whose frequent or uncritical use can dramatically harm the learning process (Bastani et al. 2025). To address these challenges, we must reimagine how we train students and researchers to engage with GenAI in ways that enhance, rather than undermine, scientific practice. This need is particularly urgent in plant science, an increasingly data rich and computationally intensive field whose complexity spans multiple biological scales—from molecular genetics and physiology to breeding and agriculture to climate modeling and ecosystem interactions. This complexity makes plant science both a fertile ground for GenAI applications and a domain where misuse can lead to significant negative scientific and societal consequences. Multiple institutions and organizations have formulated AI policies for teaching, research, and publishing, and we encourage the readers to identify policies most relevant to their context (File S1) (Bommasani et al. 2025). Here, we propose 6 strategic shifts tailored toward plant science pedagogy and suggest paths for adopting GenAI in our classrooms and labs (Fig. 1). The goal of this community-wide effort is not to convert every plant scientist into an AI specialist, but to move the discipline from limited AI engagement (uncertainty about where AI adds value, inability to evaluate or apply methods, inattentiveness toward ethical and equity implications) toward an AI-literate practice (asking the right critical questions, using AI as a learning tool, recognizing biases and ethical standards). While specific implementations of these shifts will vary based on individual context, the recommendations below present a roadmap for training our students to be critical, well-grounded applicators of AI in shaping the plant science of tomorrow.

Box 1. Review of terminology used in this article **Generative AI (GenAI):** A type of AI that learns patterns in training data and produces new data in response to input. These could be transformer-based (eg most popular LLMs) or nontransformer-based (eg generative adversarial networks, variational autoencoders, state-space models).

**LLMs:** Self-supervised machine learning models pretrained on a vast amount of text and employing billions or trillions of parameters at runtime. Commonly known examples such as ChatGPT, Gemini, Copilot, Grok, Claude, Llama, and DeepSeek are based on the transformer architecture. Small language models (SLMs) utilize millions to low billions of parameters.

**Transformers:** Introduced in 2017 and central to the popular LLMs, transformers are models that allow neural networks to understand context and relationships between different parts of the sequence using a mechanism called self-attention.

**Foundation models:** Large multiparameter GenAI models (eg LLMs) trained on vast amounts of input data, including specific biological data (https://github.com/moghelab/plant-ai-training/).

**Vibe coding:** Writing code using prompts to an LLM, without knowing and/or writing the actual syntax. The term was introduced in February 2025 by Andrej Karpathy, a co-founder of OpenAI.

**Token:** Akin to a “word” in language, a token is a single unit derived from the input to the language model, a unit generated during the model's “reasoning” processes, or a unit generated as part of the model's formal output. A token could be a set of characters in the case of a natural language (text) model, or it could be, for example, an amino acid, in the case of a protein language model.

**Embeddings:** Numerical vector representation of a given token, akin to the contextual “meaning” assigned to the token by the GenAI model. Some models, rather than generating a de novo output by processing embeddings (as LLMs do), simply output the embeddings themselves. These models are often called “embedding models”.

**Hallucination:** Linguistically coherent but factually inaccurate response generated by GenAI. Defined as “content that is nonsensical or unfaithful to the provided source content”. Language models may not only hallucinate facts but also bridge two facts through creative thinking (referred to in this article as “creative gap-filling”).

**Fine-tuning and Transfer learning:** Fine-tuning is the process of updating a pretrained language model's internal parameters using additional training data so that its outputs more closely match desired responses for specific inputs. It is the commonly used method for transfer learning, where the general knowledge a model gained during initial training is transferred and refined for a new domain or task.

**Agents:** Unlike chat models, which produce a one-off text output in response to an input, agentic AI systems are GenAI–based systems that function iteratively, can autonomously plan and generate multiple sequential outputs, and maintain state (“remember”) across iterations by incorporating previous inputs and outputs. Agentic systems combine these characteristics with the ability to autonomously select and deploy tools (such as web searches, API calls, software modules, or file read/write operations) to complete complex, goal-oriented tasks.

### Shift 1: from multidisciplinary collaborations to interdisciplinary mindset

The multidisciplinary collaborations of plant scientists have expanded in recent times, contributing to impactful solutions for complex scientific problems. However, the elevation of these interactions to interdisciplinarity and transdisciplinarity (Choi and Pak 2006; MacMynowski 2007)—frameworks that result in knowledge that is more than the sum of their parts—faces persistent structural and cultural barriers. For example, life science students face a demonstrable “math anxiety” (Perry 2004; Wachsmuth et al. 2017) while engineers lack sufficient biological context. A biologist's desire for hypothesis-driven experimental designs, mechanistic explanations, and an appreciation of biological/technical variability may conflict with an engineer's desire for iterative design-build-test-learn cycles, result optimization, and an appreciation of model trade-offs (Poser 1998; Guzey et al. 2019). Obtaining “bilingual fluency” also requires both opportunity and motivation. For cultivating an interdisciplinary mindset, it is essential that plant scientists take the lead in dismantling these silos through integrated and collaborative teaching-training activities.

Mandatory statistics and coding classes, co-taught classes, and cross-disciplinary seminars have historically been the starting points for seeding an interdisciplinary mindset. However, LLMs present an unprecedented opportunity in this regard. They present a vast store of knowledge at the students’ fingertips, being able to simplify a wide range of technical and conceptual topics, break down nuances, guide researchers step-by-step in gaining fluency in disciplines outside their immediate area of expertise, and in constructing complex experimental designs. Students are already using LLMs as a tool in their learning process. Rather than resisting this trend, educators should leverage LLMs deliberately to embed math, coding, and engineering concepts within plant course curricula (File S2). Studies have shown that LLMs can boost self-directed learning and metacognition, if used appropriately (File S3) (Lowry et al. 2025). From a connectivist pedagogical standpoint (File S3), use of digital tools such as games and online learning modules has already been shown to boost integration of complex concepts, and GenAI is not an exception in this regard (Liang and Bai 2025). While LLMs cannot simply be assumed to be reliable, their unparalleled ability to tailor their output to the learner's pre-existing level of understanding could be particularly valuable to students experiencing math anxiety (Arnett and Van Horn 2009; Madlung et al. 2011) or to students who are unable to take statistics/coding classes. For example, an LLM query such as “*I am a 2nd year plant biology undergraduate student. Please explain LASSO regression to me in simple terms that I can understand.*” may produce an example with “optimal inputs for tomato cultivation” or “gene features important for predicting function” instead of “house prices” typically cited in other instructional content. Improved LLM engagement may also enable further exploration of limitations, alternative techniques, use cases, and coding workflows. Illustrative examples of such GenAI integration in a variety of topical classes—from molecular biology and biochemistry to ecology and weed science—are provided in File S2. Explicit, educator-directed integrations are crucial starting points to foster an interdisciplinary mindset that enables plant scientists to more effectively connect with experts in other disciplines.

We emphasize that while LLMs can simplify interdisciplinary concepts, they should not be considered as a replacement to expert domain knowledge. They should complement, not substitute (Lehmann et al. 2025). LLMs should act as a catalyst for interdisciplinary collaborations, and not as an agent for new silos. It is also important to recognize that there is no “one-size-fits-all” approach. Indeed, there are at least 3 groups of plant scientists, each requiring different types of AI training: (i) Generalists, who are plant specialists but need general literacy in querying language models (Box 1) effectively and in understanding the caveats of the output, (ii) Power users, who may be plant specialists or engineers who wish to code, automate analyses, or develop advanced workflows, and (iii) Developers, who may be engineers or plant specialists able to build and fine-tune complex model architectures for addressing plant science problems. Early course-embedded and LLM-enabled exposure to math, coding, engineering, and plant science concepts may facilitate mobility across these groups and encourage an interdisciplinary mindset.

### Shift 2: from vibe-based approaches to knowledge-based thinking

Until recently, advanced data analysis within research groups was the domain of bioinformatics specialists. However, “vibe coding” (Box 1) enables anyone to write sophisticated code with detailed annotations in any programming language seamlessly, with a few prompts. Indeed, a recent Gemini-based AI system was demonstrated to generate workflows for complex tasks like single-cell RNA-seq analysis through simple prompting that surpassed state-of-the-art tools (Aygün et al. 2026). Students are already designing complex scripts for data analysis with LLM help, often without any coding experience or the ability to manually troubleshoot issues in the code. Considering that LLMs can be used to summarize documents, generate figures, formulate hypotheses, and more, these considerations are important not only for coding but also more generally for scientific research practices. Akin to vibe coding, “vibe research” is expected to be on the rise, where individuals with limited experience will be able to generate seemingly convincing scientific arguments, protocols, workflows, illustrations, and ideas without an understanding of what is generated (Down 2025).

While vibe coding is very powerful, it leads to cognitive offloading (Fawzy et al. 2025), which may liberate the researcher from worrying about syntax but can also be a concerning source of errors. Users may skip or overlook quality assessment practices and testing, relying again on AI to validate the coded workflows. A user's lack of understanding of essential programming syntax and biases (Box 2; File S4, Case 6) can further exacerbate this behavior and can cause excessive dependency on the LLM. If the LLM is not correctly aligned to the user's logic stream or if the researcher makes mistakes in prompting, the LLM can create erroneous logic of its own or set inappropriate default parameters deep inside the code (File S4, Case 6). If the researcher does not understand the code, it is possible that a mistake will never be detected, causing significant problems downstream. The same concerns also hold true for vibe research, where the experimenter has not thought deeply about the experimental design.

Box 2. LLMs and differentiating fact from fiction **How LLMs work**: LLMs are probabilistic prediction models that—based on their training data and settings—predict the likely next token in a response. They achieve contextual understanding by virtue of being trained on vast amounts of data, complex self-attention-based architectures, and ability to perform internal checks through reasoning.

**Variations in LLM responses**: LLM responses may vary due to their training data, developer-chosen parameter settings, their “temperature”—which adjusts the creativity of their responses—instruction tuning, token limit, memory of previous conversations, etc. An LLM “hallucination” occurs when the model generates linguistically coherent but factually incorrect content, such as invented gene names, fake pathways, or nonexistent papers. Bias can appear as overconfidence in model organisms, preference for dominant theories, or reinforcement of popular but untested claims. Examples are listed in File S4.

**Differentiating fact from fiction**: Lack of uncertainty language, use of unusual style words, exaggerations, missing or unverifiable citations, vague experimental descriptions, sycophantic behavior, and claims that cannot be traced to primary literature are major red flags. Cross-questioning the LLM, listing assumptions and limitations, asking secondary probing questions, asking the same question multiple times, or to different LLMs may reveal the stable elements of a response (File S5). Ultimately, LLMs should be treated as hypothesis generators—the student/researcher must be responsible for the final synthesis.

Due to these serious consequences of vibe coding and vibe research, we propose a 4-pronged approach: *(i) Teach empowering logical thinking skills:* Abilities to connect the dots through deductive/inductive thinking are crucial to foster and emphasize. In coding, for example, essential programming syntax (data structures, iterations, control flow, conditional logic), engineering logic (unit/integration testing) and good prompting skills should be made a mandatory component of all plant science curricula—along with an understanding of ethical issues in AI (see Shift 5)—to empower critical engagement of students and researchers with GenAI over its passive acceptance. This is an essential step in transitioning the burgeoning number of code-writing plant scientists to be effective troubleshooters. Such instruction could be implemented as a set of lessons embedded within existing courses, a short curriculum module, or a one-credit course/workshop explicitly focused on coding/vibe coding. *(ii) Emphasize scientific first principles:* While GenAI can accelerate hypothesis generation and interpretation, it cannot replace experiments or the datasets they produce. Student training should emphasize the invariant foundations of science, such as falsifiability, controls, hypothesis testing, and so forth, as well as the irreplaceability of domain-specific knowledge. Rather than focusing narrowly on procedural tasks eg “*how to generate a PCA plot in R*” or factual information eg, “*what are the reaction steps in a biosynthetic pathway*”—teaching underlying concepts such as dimensionality reduction, clustering logic, and their mathematical basis, or overall pathway logic using a multidisciplinary perspective (File S2, Activity B)—can position students on foundations that are stable to changes in GenAI capabilities. *(iii) Build resiliency:* Educators should encourage students to unlearn any cognitive dependence on LLMs and build *their own* scientific identity before offloading thinking to an LLM. Writing exercises can be incorporated to develop critical thinking skills (Editorial 2025; Kosmyna et al. 2025) and an appreciation of the students’ own biases and limitations. Students could also be asked to solve problems combining multiple modes of analysis—manual reasoning, command-line tools, and AI-assisted workflows. For example, a botany educator may ask students to identify 10 plant species using a dichotomous key, an image reverse search, an identification app, and a multimodal LLM (File S2, Examples C, E). Explicit comparisons of outputs across approaches can reveal hidden assumptions, strengths, and weaknesses of the workflows. *(iv) Foster tool independence and modular workflow design:* As the time to code decreases and AI-developed software (Aygün et al. 2026) proliferates, rigid workflows will quickly become obsolete (File S4, Cases 6 and 8). Teaching modular experimental design with swappable steps and tools can help the students absorb the overall task logic, promote conceptual understanding, reveal tool biases and limitations, and make students more critical, adaptive, and creative about tooling and research practices.

### Shift 3: from restricting AI use in classrooms to embracing it

According to a 2025 survey (Flaherty 2025), 85% of the US college students surveyed used GenAI in the previous year. One legitimate response has been to restrict GenAI use in classes, driven by fears of cheating, plagiarism, and a compromised learning process (Bastani et al. 2025). Instructors themselves may not be conversant enough in the biases and limitations of GenAI, making them reluctant to allow its usage. Other factors contributing to the desire to restrict its use include a lack of trust in GenAI responses’ accuracy and reliability, privacy concerns in how training data is being collected, negative media representation due to cases of misuses, challenges in understanding how the responses are generated, and concerns of being replaced (Caballar 2024; Rogers 2025). Many institutions have already articulated GenAI-use policies; however, the ubiquity of LLMs, their human-like responses, and their integration into learning platforms makes any restrictions difficult to enforce. Furthermore, strict LLM policies may also end up penalizing students who have put effort and skill into the assigned work (Carruba et al. 2025). From the perspective of upskilling students, some universities have already deemed AI fluency as an important requirement for graduation, and it is imperative that such fluency also integrates field-specific training for plant science students.

Our illustrative examples (Files S2, S4, and S5) and a large compilation of AI-relevant online workbooks (https://github.com/moghelab/plant-ai-training) highlight how some existing lectures can be adapted for the GenAI era. Classroom instruction could also include concrete examples of using iterative conversations with an LLM—interspersed with non-LLM-based instruction—to learn a new concept until understanding is achieved. The key message must be that GenAI should be used to facilitate but not to dominate the learning process. It should not complete tasks for learners but rather support them in curiosity-driven exploration.

For effective evaluation of student learning, approaches including in-person assessments, interviews, in-class timed quizzes, and participation-based grading have withstood the test of time and may be applicable based on class scope and size. Additional LLM-era solutions include: *(i) Process-based assessment*, where the LLM prompts are graded, not the outcome, assessing whether the student exhibited an understanding of the problems and developed effective subsequent prompts (Hu 2025). For example, an acknowledgment statement such as “*I used Copilot to draft the image segmentation code, but I modified the thresholding logic because the AI's suggestion conflated drought stress with senescence. I validated using this manual subset…*” is sufficiently indicative of the student's grasp of the subject matter. *(ii) Explicit AI integration*, where students compare and contrast different LLM responses, can be used to assess whether they showed an understanding of the variations and biases of the responses. Conversely, students can be provided GenAI-generated content (code, text, images, protein structures, molecules, etc.) and be asked to evaluate it to demonstrate their learned knowledge and expertise (Files S2 and S4). For example, *can LLMs accurately infer Mendelian genetics from Mendel's own data?* (File S2, Activity A) *(iii) AI-enabled student projects:* Individually or in teams, students can be asked to develop projects, explore datasets, develop and implement modular workflows, and interpret and present outcomes—all with GenAI assistance as needed. Explicit requirements for documentation, reproducibility, and human-in-the-loop validation will assure engagement of the students with an understanding of the challenge and the solution. Treating GenAI-generated outputs as hypotheses rather than conclusions reinforces experimental rigor while leveraging GenAI for rapid iteration. The research concept, project management, execution, and communication skills can be graded.

### Shift 4: from casual use of GenAI to ensuring biases and hallucinations are mitigated

Given LLMs can be sycophantic (Cheng et al. 2026) or sometimes respond with unbridled confidence, it is important that students know how GenAI works, how biases and hallucinations originate (File S4: Case 1 and Case 8), the types of such errors in plant science context (Box 2; File S4), and how to mitigate them. For example, querying LLMs for “*plants that resprout after fire*” gave different species lists for English vs. Spanish queries (File S4: Case 5). In one study involving LLM-based extraction of plant enzyme-substrate interactions from papers (Smith et al. 2025), the model hallucinated entries from human and rat species in DrugBank, likely because of its training data bias. Probing an LLM response on phylogenetic reasoning further with a follow-up self-critique prompt resulted in variant and sometimes incorrect responses by 3 different LLMs (File S4: Case 2). The same AI tool produced different metabolite networks when prompted with the same spectral data 3 months apart (File S4: Case 8). While the rapid advances in GenAI are expected to reduce the frequency of such mistakes, it will be challenging to eliminate them. Nonetheless, these are serious scientific errors that first need to be recognized before time and resources are dedicated to downstream validation.

One might argue that in most cases, bias is due to an uneven representation of training data, such as in species represented. Biomedically relevant and genetically tractable species with large research communities, model systems (eg Arabidopsis, tomato, rice, maize), temperate species, diploids, conspicuous/charismatic species (Guénard et al. 2025; Tensen and Teske 2025), commodity crops, and species from richer countries are likely to be overrepresented in AI training data (Marks et al. 2023) (File S2). Therefore, an AI model trained to predict stress responses from Arabidopsis, maize, and rice could be dangerously inaccurate when applied to tropical crops like mango or pigeon pea. Similarly, AI solutions may be optimized for productivity or maximum yield and applicable for large farms practicing monoculture, but may fail and exacerbate inequality for farmers in the low- and middle-income countries, who typically practice smallholder, diversified agriculture with risk minimization or water use efficiency as critical metrics. From a technical perspective, ecological data is more likely to be collected on roadsides and phenotypic data in growth chambers, greenhouses, and highly controlled in vitro conditions, creating data distributions that may not translate to a user's specific context. Systematic social inequalities such as gender biases (Newstead et al. 2023; Nganga et al. 2025) may also be baked into and further perpetuated by predictive infrastructures (Obermeyer et al. 2019). Documenting and accounting for considering data biases in national agricultural statistics and broader agricultural research data infrastructure could avoid locking these biases into agricultural policies. While most popular LLMs are multilingual, there is a notable bias toward English language training data (Pava et al. 2025; Yao et al. 2025), and indigenous knowledge may not be accessible with normal prompts (Perera et al. 2025). Furthermore, the rise of AI slop in news articles, forum posts, media content, and journal articles (Elali and Rachid 2023; Down 2025; Jones et al. 2025; Kusumegi et al. 2025) exacerbates the challenge of differentiating true knowledge from AI-generated and hallucinated content.

Better generation and selection of the training data, prompt engineering, better reasoning, fine-tuning, and the use of retrieval augmented generation (File S5) may mitigate or overcome some of these biases and issues with hallucination. Nonetheless, given the vast black box that GenAI is, we emphasize 3 core practices: *(i) Discuss AI biases in the classroom*, explicitly in introductory lectures, training students to make informed decisions about when GenAI use is appropriate, weighing trade-offs between efficiency and risk. As an active learning process, students, for example, can be made to research, list and provide examples of biases they might encounter in using a DNA foundation model (Box 2) *(ii) Question the output:* LLMs may engage in creative gap filling, hallucinations (Box 2, File S4: Cases 1 and 8) and sycophantic behavior designed to please the user rather than uncover the truth (Cheng et al. 2026), necessitating critical assessment. Indeed, skepticism and the ability to design optimal and follow-up questions are some of the most important skills to be learned in the GenAI era. *(iii) Validate responses:* As GenAI expands from simple LLMs to agentic AI and varied biological foundation models, some of which are easily accessible to nonspecialists in a chat-like interface (math-gpt.org; openbio.tech), students must learn to rigorously question and validate them. For LLMs, at least 6 different types of validation methods have been proposed (File S5), some of which are straightforward to embed in coursework (File S2). The plant science curriculum must instill technical literacy in how AI models are evaluated (Mahood et al. 2020). Knowledge graph (Gaur et al. 2021; Rajabi and Etminani 2024) (File S5) and probing-perturbing-surrogate strategies (Azodi et al. 2020), for example, are useful tools for biologists to mitigate the black box nature of GenAI and enable uncertainty quantification by revealing the basis of the GenAI predictions. Such training can help ensure the correct application of AI in plant sciences. We reemphasize, however, that having strong foundational knowledge in the core subject and critical thinking skills is essential for any AI practitioner to detect such hallucinations.

### Shift 5: encouraging ethical and responsible AI use

In plant sciences, GenAI introduces unique ethical challenges that go beyond the general paradigm of academic integrity (Eitel-Porter 2021; ERA 2025). For example, while many models are free or offer a free academic use tier, the best LLMs are typically available with a subscription or may charge API access fees, which is prohibitive for many labs, universities, and countries—further exacerbating the divides between the haves and the have-nots. Decisions about which data are included, how they are curated, and how models are fine-tuned are made by a relatively small group of people. The choice these people make could exacerbate existing biases about what constitutes valid sources and knowledge. Students should recognize that the models reflect the values, priorities, limitations, and biases of their creators.

Another issue relates to data sovereignty. Indiscriminate scraping of copyrighted web data for training LLMs has been criticized earlier (Fontana 2025; Russ-Smith and Randell-Moon 2025), and when researchers upload their ideas, data, and images into the chatbots or make API calls to models, they must be aware of how this data is used. For example, queries and responses on the free tier of every LLM are used for model training and/or human oversight. Cognizance about data security and sovereignty is especially critical in industry, industry-academia collaboration, national security settings, and in relation to indigenous knowledge systems (Russ-Smith and Randell-Moon 2025). While AI can fortify data security, it may also lead to unprecedented and unpredictable data and privacy breaches. Researchers using coding assistants and sharing private data and figures with LLMs run the risk of intellectual property breaches, which in some cases may constitute research misconduct. AI practitioners therefore should be aware of best practices for data security (Junior and Clinton 2025; National Academies of Sciences, Engineering, and Medicine). Passing dummy data or only a few rows of data to LLMs, copy-pasting the Python/R script generated by the LLM into a local coding environment for execution instead of a real-time connection, protecting sensitive paths in local environments through configuration files, using local LLMs and SLMs (Busta and Oyler 2025), etc., are some security steps researchers might take to protect their data.

At larger scales, these concerns are leading to nations rushing to build their own sovereign GenAI models, not just for data security but also for more effective delivery of essential services to their citizens (Pandey 2025). Also, several intellectual property laws regarding indigenous knowledge and biodiversity, access, and benefit sharing with the communities who have stewarded these species for millennia were created before the GenAI era, and must be rethought today (Nunez-Vega et al. 2025). Legal updates cannot keep up with the fast-paced development of AI. However, if a foundation model trained on billions of data points identifies the biosynthetic genes of a drug from the digitized genome of an Amazonian medicinal plant species, do the indigenous communities benefit from the resulting product? Discussion of such AI ethics questions in plant science classes is imperative for AI literacy.

When GenAI, and AI more broadly, is used in agriculture, it creates another set of ethical and legal issues. AI-driven job losses and stagnation are real concerns. Furthermore, when AI makes mistakes in recommendations and damages crops and property (Prostko 2025), who is held accountable? For data captured through field sensors and uploaded to commercial AI platforms, who owns the data (Dara et al. 2022)? Due to the above-discussed biases and limitations, GenAI-enabled chatbots—which can be transformational for knowledge access to farmers—can also be a source of agricultural misinformation (Silva et al. 2023). Lack of explainability, accountability, transparency, and questionable data ownership may erode farmer confidence in AI platforms (Mark 2019; Thomasson 2025), or worse, be a source of farmer distress. Students, whether interested in academia, industry, or policy careers, should be cognizant of these issues.

An additional concern is that widespread LLM use may be leading to an increasing consistency of responses and a loss of alternative perspectives. For example, LLMs tend to use specific code and writing styles. One study demonstrated that style words such as “delves,” “notably,” “pivotal,” and others showed an unprecedented increase in biomedical abstracts in 2024—2 years after ChatGPT was released (Kobak et al. 2025). LLM-generated workflow figures that follow a standard representational format are increasingly commonplace. Such an inadvertent trend toward uniformity is harmful for human creativity (Kumar et al. 2025). The dominant perspectives may be amplified, while unconventional or emerging ideas (especially those of underrepresented regions, languages, or disciplines) could become less visible. Innovation often arises from studying unusual systems, extreme environments, and locally adapted species, so this homogenization could have negative effects. LLMs may limit exploration of alternative ideas, approaches, and taxa, which would reduce intellectual diversity rather than expanding it.

LLMs—especially the models that reveal the chain-of-thought process—can further create an “illusion of thinking” (Shojaee et al. 2025), which may translate to an illusion of competence on part of the student/researcher, not only harming the development of critical thinking skills but also scientific rigor. The Cognitive Load Theory (File S3) suggests that integration of GenAI should reduce the intrinsic and extraneous loads of learning and free up the working memory resources available for the germane load—referring to the efforts needed to convert short-term information to long-term understanding (Sweller 2023). However, if LLMs reduce the germane load, students may feel like they have learned a concept without actually doing so (Bai et al. 2023). Indeed, one study found that students with less initial knowledge of the core topic learned less when using LLMs compared to students with high initial knowledge, exacerbating classroom inequality (Lehmann et al. 2025). These outcomes are undesirable for the trustworthiness and sustainability of the scientific enterprise built largely on taxpayer-funded research.

Finally, a less-discussed consequence of LLMs is the risk of decoupling knowledge production from lived experience, place, and human interactions. Plant science has long depended on fieldwork and sustained engagement with people who live and work in the ecosystems we study (eg farmers, land managers, community scientists, and herbarium curators). Much of the learning in plant science classes occurs in farms, gardens, and wild trails. Greater LLM use may create an illusion of knowledge and wrongly devalue these interpersonal interactions and experiences. This will reduce opportunities for experiential learning, community building, in-person collaboration, and dialogue across cultures, which are key for building equitable partnerships.

We advocate for the incorporation of ethical and responsible use training into any AI-related coursework, which could include a focus on any of the topics of concern mentioned above. Most importantly, AI applicators need to be honest about when and how AI is used, and educators need to create appropriate frameworks for reporting this information. This is also important to emphasize the importance of transparency when communicating research findings—particularly how GenAI is used in developing the ideas, designing experiments, analyzing results, and drafting manuscripts.

### Shift 6: from local resources to regional training infrastructures

The shifts described above range from simple actions implementable at an individual level to those requiring access to specialized resources. However, training students in using AI in resource-constrained institutions and low- and middle-income countries presents a range of challenges rooted in infrastructure, data access, and capacity. Technical barriers such as limited computational resources, poor internet connectivity, unreliable electricity supply, and high cost of GPUs make it difficult to train, deploy, or access AI systems effectively in many institutional settings. Many of the world's biodiversity hotspots and regions facing acute agricultural challenges are also those with the least private access to advanced computing systems. All these issues are compounded by a shortage of skilled personnel—researchers and educators—who can help create an AI-literate plant science workforce. Frequently, for many educators, knowing where to start learning and teaching AI itself is the first challenge.

To address these issues stymying the adoption of AI in plant science research, we propose 3 critical solutions: *(i) Empower regional supercomputing infrastructures:* In several geographies, pay-as-you-go commercial providers such as Google Cloud, Microsoft Azure, and AWS are an effective solution for lack of institutional access. However, globally, governments have also invested in HPC resources where researchers from that region can access supercomputing capacity free of cost. A long list of such regional infrastructure available for requesting compute power, data hosting, and user training is presented in File S6. We imagine a scenario where a researcher working on plant-pathogen interactions in a remote university is able to fine-tune a global “PlantVision” foundation model on her local disease phenotypes using the nearest national AI-network hub, contributing her data back to a shared global repository. Such seamless and global data contribution and data access can turbocharge development of highly impactful, globally applicable genotype-to-phenotype models in plant sciences and make the community more adaptive to emerging threats. We encourage plant scientists to utilize these resources for hands-on learning, and advocate for more public investment and equitable access to these infrastructures. *(ii) Promote FAIR-compliant data systems and middleware:* The scale of plant science—from genes to organisms to ecosystems—is a boon for multimodal data gathering. Unfortunately, the field lacks a unified, cross-species infrastructure comparable to The Cancer Genome Atlas or ENCODE. The different formats of captured data—from hand-scribbled field notes to proprietary files—are poorly accessible through APIs, labor-intensive to reformat, and of limited utility in AI model development despite access to the best supercomputing facilities. There are limited ready-to-use datasets available for educators to build AI tutorials in different fields of plant science, and finding plant-specific datasets and tutorials on general-purpose databases such as Kaggle is also not an easy task. We propose here that consortia (eg Plant Cell Atlas, AgBioData, BrAPI), databases (eg Phytozome, Gramene, GRIN, Ensembl, FAOSTAT) and plant science societies/journals take the lead in strengthening existing frameworks established by ELIXIR, Research Data Alliance etc., help develop global FAIR-compliant repositories for plant multimodal data, and ensure public access to taxpayer-generated datasets. Furthermore, support for no-code interfaces and middleware such as Galaxy, ePlant, plantscience.ai, PlantConnectome, PMN, and Plant Reactome is also crucial to ensure that command-line expertise does not become a gatekeeper for AI adoption. These changes will not only enhance model generalizability and utility but also ensure that students are trained in deploying AI pipelines effectively on a diversity of domain-specific datasets, species and environments. *(iii) Create national and global AI training programs:* In the AI space, there is a plethora of free software and step-by-step tutorials for self-guided learning available on the internet, but most of it does not use plant-relevant examples, impeding learning and innovation. Plant science educators can create a backbone of training resources geared toward equitable learning across geographies. While our list of activities (File S2) and a compilation of online resources (https://github.com/moghelab/plant-ai-training/) is a start, development of tutorials on deploying AI models on topics relevant to breeding, plant biochemistry, image analysis, ecology etc. can help bridge institutional resource gaps and foster computational literacy for plant scientists even in low-infrastructure environments. Development of classroom pedagogy should closely align with emerging wisdom and educational frameworks (File S3). For example, The Bicycle Principles (Williams et al. 2023) provide learner-centered guidance that applies outside the formal classroom context, specifically, short-format training. The Principles emphasize preparing learners with clear prerequisites, authentic tasks, and pathways for continued practice. Such well-informed training programs—not just for students but also for educators—can be implemented in collaboration with regional HPC hubs and national plant science societies, and be made available online freely and centrally for global access. Wherever possible, physical presence in labs and classrooms through plant-focused course-based undergraduate research experiences, summer research internships, industry-academia collaborations, and other mechanisms can also build the interpersonal connections required for an adaptive, innovative, and responsible AI-forward plant science community.

## Concluding remarks

In conclusion, we reemphasize that across the diversity of plant sciences, we must ensure that teaching and mentoring practices keep pace with the rapid buildup of AI and GenAI capabilities. While AI is a powerful tool to dramatically accelerate research and learning, the wisdom to use it correctly depends on the strength of foundational subject knowledge, critical thinking skills, ability to ask good questions, and appropriate use of the scientific method—skills that are very difficult to self-learn despite GenAI, thereby reinforcing the value of a rigorous college education. While prioritizing innovation in plant sciences by embracing AI's transformative potential, we must make active efforts to ensure that we do not sacrifice the lived experience, cultural diversity, and experiential learning that have long been the discipline's foundation. Our challenge is to build a generation of plant scientists who are critical and responsible practitioners of AI-enabled science and who can harness AI technologies as one of the many tools to address the urgent challenges of food security, climate adaptation, and biodiversity conservation. Furthermore, as a global community of researchers and educators, we must create and support infrastructure and emerging wisdom that democratizes AI's transformative potential for complex plant science challenges. The six shifts we propose here constitute a broad, initial framework for integrating GenAI into plant science teaching and mentoring. With the rapidly evolving landscape of AI capabilities and limitations, we expect this framework will also need ongoing adaptation.

## Supplementary Material

koag140_Supplementary_Data

## Data Availability

The data underlying this article are available in the Supplementary Material and at the following GitHub link: https://github.com/moghelab/plant-ai-training.

## References

[koag140-B1] Arnett A, Van Horn D. 2009. Connecting mathematics and science: a learning community that helps math-phobic students. J Coll Sci Teach. 38:30–34. https://www.jstor.org/stable/42993314.

[koag140-B2] Aygün E et al 2026. An AI system to help scientists write expert-level empirical software. Nature. 10.1038/s41586-026-10658-6. PMC13293872 42156545

[koag140-B3] Azodi CB, Tang J, Shiu S-H. 2020. Opening the black box: interpretable machine learning for geneticists. Trends Genet. 36:442–455. 10.1016/j.tig.2020.03.005. 32396837

[koag140-B4] Bai L, Liu X, Su J. 2023. ChatGPT: the cognitive effects on learning and memory. Brain-X. 1:e30. 10.1002/brx2.30.

[koag140-B5] Bastani H et al 2025. Generative AI without guardrails can harm learning: evidence from high school mathematics. Proc Natl Acad Sci U S A. 122:e2422633122. 10.1073/pnas.2422633122. 40560616 PMC12232635

[koag140-B6] Bommasani R et al 2025. Advancing science- and evidence-based AI policy. Science. 389:459–461. 10.1126/science.adu8449. 40743343

[koag140-B7] Busta L, Oyler AR. 2025. Small language models enable rapid and accurate extraction of structured data from unstructured text: an example with plants and their specialized metabolites. Quant Plant Biol. 6:e26. 10.1017/qpb.2025.10021. 40989012 PMC12451238

[koag140-B8] Caballar R. 10 AI dangers and risks and how to manage them | IBM. IBM. 2024. https://www.ibm.com/think/insights/10-ai-dangers-and-risks-and-how-to-manage-them. Retrieved February 10, 2026

[koag140-B9] Carruba MC, Caiazzo A, Scuotto C, Savioni L, Triberti S. 2025. A grade for artificial intelligence: a study on school teachers’ ability to identify assignments written by generative artificial intelligence. Cyberpsychol Behav Soc Netw. 28:489–496. 10.1089/cyber.2024.0524. 40471066

[koag140-B10] Cheng M et al 2026. Sycophantic AI decreases prosocial intentions and promotes dependence. Science. 391:eaec8352. 10.1126/science.aec8352. 41886588

[koag140-B11] Choi BCK, Pak AWP. 2006. Multidisciplinarity, interdisciplinarity and transdisciplinarity in health research, services, education and policy: 1. Definitions, objectives, and evidence of effectiveness. Clin Invest Med. 29:351–364. https://pubmed.ncbi.nlm.nih.gov/17330451/. 17330451

[koag140-B12] Dara R, Hazrati Fard SM, Kaur J. 2022. Recommendations for ethical and responsible use of artificial intelligence in digital agriculture. Front Artif Intell. 5:884192. 10.3389/frai.2022.884192. 35968036 PMC9372537

[koag140-B13] Down A . 2025. Artificial intelligence research has a slop problem, academics say: ‘It's a mess’. The Guardian.

[koag140-B14] Editorial . 2025. Writing is thinking. Nat Rev Bioeng. 3:431–431. 10.1038/s44222-025-00323-4.

[koag140-B15] Eitel-Porter R . 2021. Beyond the promise: implementing ethical AI. AI Ethics. 1:73–80. 10.1007/s43681-020-00011-6.

[koag140-B16] Elali FR, Rachid LN. 2023. AI-generated research paper fabrication and plagiarism in the scientific community. Patterns. 4:100706. 10.1016/j.patter.2023.100706. 36960451 PMC10028415

[koag140-B17] ERA . Living guidelines on the responsible use of generative AI in research | Research and innovation. 2025. https://research-and-innovation.ec.europa.eu/document/2b6cf7e5-36ac-41cb-aab5-0d32050143dc_en. Retrieved February 10, 2026

[koag140-B18] Fawzy A, Tahir A, Blincoe K. 2025. Vibe coding in practice: motivations, challenges, and a future outlook -- a Grey Literature Rev. arXiv. 10.48550/arXiv.2510.00328.

[koag140-B19] Flaherty C. How AI Is Changing—Not ‘Killing’—College. Inside Higher Ed. 2025. https://www.insidehighered.com/news/students/academics/2025/08/29/survey-college-students-views-ai. Retrieved January 25, 2026.

[koag140-B20] Fontana A . 2025. Web scraping: jurisprudence and legal doctrines. J World Intellectual Property. 28:197–212. 10.1111/jwip.12331.

[koag140-B21] Gaur M, Faldu K, Sheth A. 2021. Semantics of the Black-Box: can knowledge graphs help make deep learning systems more interpretable and explainable? IEEE Internet Comput. 25:51–59. 10.1109/MIC.2020.3031769.

[koag140-B22] Guénard B et al 2025. Limited and biased global conservation funding means most threatened species remain unsupported. Proc Natl Acad Sci U S A. 122:e2412479122. 10.1073/pnas.2412479122. 39993186 PMC11892620

[koag140-B23] Guzey SS, Ring-Whalen EA, Harwell M, Peralta Y. 2019. Life STEM: a case study of life science learning through engineering design. Int J Sci Math Educ. 17:23–42. 10.1007/s10763-017-9860-0.

[koag140-B24] Hu X. Assessment in the AI Era: Grade the thinking, not just the text. University of Arizona News. 2025. https://news.arizona.edu/employee-news/assessment-ai-era-grade-thinking-not-just-text. Retrieved January 25, 2026

[koag140-B25] Jones EM, Newman JD, Kim B, Fogle EJ. 2025. AI-generated “slop” in online biomedical science educational videos: mixed methods study of prevalence, characteristics, and hazards to learners and teachers. JMIR Med Educ. 11:e80084. 10.2196/80084. 41264860 PMC12634010

[koag140-B26] Junior E, Clinton S. 2025. LLM and GenAI data security best practices: OWASP top 10 for LLM Apps and Gen AI data security initiative.

[koag140-B27] Kobak D, González-Márquez R, Horvát E-Á, Lause J. 2025. Delving into LLM-assisted writing in biomedical publications through excess vocabulary. Sci Adv. 11:eadt3813. 10.1126/sciadv.adt3813. 40601754 PMC12219543

[koag140-B28] Kosmyna N et al 2025. Your brain on ChatGPT: accumulation of cognitive debt when using an AI assistant for essay writing task. 10.48550/arXiv.2506.08872.

[koag140-B29] Kumar H, Vincentius J, Jordan E, Anderson A. 2025. Human creativity in the age of LLMs: randomized experiments on divergent and convergent thinking. Proc 2025 CHI Conf Hum Factors Comput Syst 1–18. 10.1145/3706598.3714198.

[koag140-B30] Kusumegi K et al 2025. Scientific production in the era of large language models. Science. 390:1240–1243. 10.1126/science.adw3000. 41411417

[koag140-B31] Lehmann M, Cornelius PB, Sting FJ. 2025. AI meets the classroom: when do large language models harm learning? arXiv. 10.48550/arXiv.2409.09047.

[koag140-B32] Liang ES, Bai S. 2025. Generative AI and the future of connectivist learning in higher education. J Asian Public Policy. 18:329–351. 10.1080/17516234.2024.2386085.

[koag140-B33] Lowry B, McGrath S, Eitel C, Hall H, Clapp TR. 2025. Leveraging generative AI to foster metacognition and self-directed learning. J Microbiol Biol Educ. 27:e0015325. 10.1128/jmbe.00153-25. 41294341 PMC13131016

[koag140-B34] MacMynowski DP . 2007. Pausing at the brink of interdisciplinarity: power and knowledge at the meeting of social and biophysical science. Ecol and Soc. 12. https://www.jstor.org/stable/26267854.

[koag140-B35] Madlung A, Bremer M, Himelblau E, Tullis A. 2011. A study assessing the potential of negative effects in interdisciplinary math–biology instruction. CBE—Life Sci Educ. 10:43–54. 10.1187/cbe.10-08-0102. 21364099 PMC3046887

[koag140-B36] Mahood EH, Kruse LH, Moghe GD. 2020. Machine learning: a powerful tool for gene function prediction in plants. Appl Plant Sci. 8:e11376. 10.1002/aps3.11376. 32765975 PMC7394712

[koag140-B37] Mark R . 2019. Ethics of using AI and big data in agriculture: the case of a large agriculture multinational. ORBIT J. 2:1–27. 10.29297/orbit.v2i2.109.

[koag140-B38] Marks RA et al 2023. A critical analysis of plant science literature reveals ongoing inequities. Proc Natl Acad Sci U S A. 120:e2217564120. 10.1073/pnas.2217564120. 36853942 PMC10013813

[koag140-B39] math-gpt.org . https://math-gpt.org. Retrieved January 25, 2026.

[koag140-B40] National Academies of Sciences, Engineering, and Medicine . The Age of AI in the Life Sciences: Benefits and Biosecurity Considerations 2025. %20Engineering, %20and%20Medicine.%202025.%20The%20Age%20of%20AI%20in%20the%20Life%20Sciences:%20Benefits%20and%20Biosecurity%20Considerations.%20Washington, %20DC:%20The%20National%20Academies%20Press.%20. 40392968

[koag140-B41] Newstead T, Eager B, Wilson S. 2023. How AI can perpetuate—or help mitigate—gender bias in leadership. Organ Dyn. 52:100998. 10.1016/j.orgdyn.2023.100998.

[koag140-B42] Nganga KG, Grossi A, Wanjau AN. 2025. Advancing gender equity in digital agro-advisory through inclusive Artificial Intelligence (AI): bias analysis and strategic recommendations from the iShamba platform based on five years of female farmers queries. AICCRA Working Paper no. Centro Internacional de Agricultura Tropical – CIAT.

[koag140-B43] Nunez-Vega G, Reimer LC, Overmann J, Scholz AH. 2025. A new indicator for the Kunming–Montreal Global Biodiversity Framework: capturing non-monetary benefit data from access and benefit-sharing agreements. BioScience. 75:298–306. 10.1093/biosci/biae132. 40276478 PMC12016788

[koag140-B44] Obermeyer Z, Powers B, Vogeli C, Mullainathan S. 2019. Dissecting racial bias in an algorithm used to manage the health of populations. Science. 366:447–453. 10.1126/science.aax2342. 31649194

[koag140-B45] openbio.tech . OpenBio. https://openbio.tech. Retrieved January 25, 2026

[koag140-B46] Pandey P . 2025. Digital sovereignty and AI: developing India's national AI stack for strategic autonomy. Procedia Comput Sci. 254:250–259. 10.1016/j.procs.2025.02.084.

[koag140-B47] Pava J et al 2025. Mind the (Language) Gap: mapping the challenges of LLM development in low-resource language contexts.

[koag140-B48] Perera M et al 2025. Indigenous peoples and artificial intelligence: a systematic review and future directions. Big Data Soc. 12:20539517251349170. 10.1177/20539517251349170.

[koag140-B49] Perry AB . 2004. Decreasing math anxiety in college students. Coll Stud J. 38:321. https://go.gale.com/ps/i.do?id=GALE%7CA119741942&sid=googleScholar&v=2.1&it=r&linkaccess=abs&issn=01463934&sw=w&u=cornell&p=AONE&aty=ip

[koag140-B50] Poser H . 1998. On structural differences between science and engineering. Soc Philos Technol Q Electron J. 4:128–135. 10.5840/techne1998426.

[koag140-B51] Prostko E. Expert warns: AI weed control advice may be incorrect and illegal. Farm Progress. 2025. https://www.farmprogress.com/crop-protection/extension-specialist-beware-of-ai-herbicide-recommendations. Retrieved January 25, 2026.

[koag140-B52] Rajabi E, Etminani K. 2024. Knowledge-graph-based explainable AI: a systematic review. J Inf Sci. 50:1019–1029. 10.1177/01655515221112844. 39135903 PMC11316662

[koag140-B53] Rogers R . 2025. The AI backlash keeps growing stronger. Wired.

[koag140-B54] Russ-Smith J, Randell-Moon H. 2025. AI and indigenous data sovereignty: knowing, engaging, and learning in new data contexts. Somatechnics. 15:287–295. 10.3366/soma.2025.0467.

[koag140-B55] Shojaee P et al 2025. The illusion of thinking: understanding the strengths and limitations of reasoning models via the lens of problem complexity. arXiv. 10.48550/arXiv.2506.06941

[koag140-B56] Silva B, Nunes L, Estevão R, Aski V, Chandra R. 2023. GPT-4 as an agronomist assistant? Answering agriculture exams using large language models. arXiv. 10.48550/arXiv.2310.06225

[koag140-B57] Smith N, Yuan X, Melissinos C, Moghe G. 2025. FuncFetch: an LLM-assisted workflow enables mining thousands of enzyme–substrate interactions from published manuscripts. Bioinformatics. 41:btae756. 10.1093/bioinformatics/btae756. PMC11734755 39718779

[koag140-B58] Sweller J . 2023. Discussion of the special issue on cognitive load theory. Br J Educ Psychol. 93:402–410. 10.1111/bjep.12606. 37160659

[koag140-B59] Tensen L, Teske PR. 2025. Research monopolization in the biological sciences: charismatic species are partly to blame. People and Nature. 7:2986–3001. 10.1002/pan3.70158.

[koag140-B60] Thomasson A. AI in Agriculture: Opportunities, Challenges, and Recommendations—CAST—The Council for Agricultural Science and Technology. 2025. https://cast-science.org/publication/ai-in-agriculture-opportunities-challenges-and-recommendations/. Retrieved January 25, 2026.

[koag140-B61] Wachsmuth LP, Runyon CR, Drake JM, Dolan EL. 2017. Do biology students really hate math? Empirical insights into undergraduate life science majors’ emotions about mathematics. CBE Life Sci Educ. 16:ar49. 10.1187/cbe.16-08-0248. 28798211 PMC5589429

[koag140-B62] Williams JJ et al 2023. An international consensus on effective, inclusive, and career-spanning short-format training in the life sciences and beyond. PLoS One. 18:e0293879. 10.1371/journal.pone.0293879. 37943810 PMC10635508

[koag140-B63] Yao Z, Duan L, Xu S, Chi L, Sheng D. 2025. Performance of large language models in the non-English context: qualitative study of models trained on different languages in Chinese medical examinations. JMIR Med Inform. 13:e69485. 10.2196/69485. 40577654 PMC12227152

