# Supplementary material for: Reimagining plant science training in the era of generative artificial intelligence: a global perspective
Source: Plant Cell. 2026 May 12;38(6):koag140. doi: 10.1093/plcell/koag140 (PMC13275178; doi:10.1093/plcell/koag140)
Supplement: koag140_Supplementary_Data [file koag140_supplementary_data.pdf]

Supplementary File 1: Relevant guidelines and advisories by organizations

All links were accessed in January 2025

A. Policy documents

AI in agriculture: Opportunities, Challenges and Recommendations
https://cast-science.org/publication/ai-in-agriculture-opportunities-challenges-and-recommendations/

Centre for Agriculture and Bioscience International (CABI) - Generative AI for Agriculture Advisory
https://www.cabi.org/projects/generative-ai-for-agriculture-advisory/

Center for International Governance Innovation (CIGI) - Generative AI's Copyright Challenges in Agricultural Extension
https://www.cigionline.org/publications/generative-ais-copyright-challenges-in-agricultural-extension/

Guidelines on the responsible use of generative AI in research developed by the European Research Area Forum
https://research-and-innovation.ec.europa.eu/news/all-research-and-innovation-news/guidelines-responsible-use-generative-ai-research-developed-european-research-area-forum-2024-03-20_en

National Science Foundation (NSF) generative AI policy
https://www.nsf.gov/news/notice-to-the-research-community-on-ai

Supporting Fairness and Originality in NIH Research Applications
https://grants.nih.gov/grants/guide/notice-files/NOT-OD-25-132.html

Living guidelines on the responsible use of generative AI in research
https://research-and-innovation.ec.europa.eu/document/2b6cf7e5-36ac-41cb-aab5-0d32050143dc_en

UNESCO - Guidance for generative AI in education and research
https://www.unesco.org/en/articles/guidance-generative-ai-education-and-research

US - Department of Education: Artificial Intelligence and the Future of Teaching and Learning
https://www.ed.gov/sites/ed/files/documents/ai-report/ai-report.pdf

South African National AI policy framework of 2024
https://www.dcdt.gov.za/sa-national-ai-policy-framework/file/338-sa-national-ai-policy-framework.html

National Institute of Standards and Technology (NIST) AI Risk Management Framework
https://www.nist.gov/itl/ai-risk-management-framework
Commission on Publication Ethics (COPE): Authorship and AI tools
https://publicationethics.org/guidance/cope-position/authorship-and-ai-tools
https://publicationethics.org/cope-focus/cope-focus-artificial-intelligence

B. Skills and competencies

Plant Science competencies: The Next Generation of Training for Arabidopsis Researchers: Bioinformatics and Quantitative Biology
https://academic.oup.com/plphys/article/175/4/1499/6116819

Ten simple rules for using large language models in science, version 1.0
https://journals.plos.org/ploscompbiol/article?id=10.1371/journal.pcbi.1011767

International Society for Computational Biology (ISCB) competency framework
https://academic.oup.com/bioinformaticsadvances/article/4/1/vbae166/7903279

ETH Zurich - Ethical Prompting for Generative AI
https://mpb.ethz.ch/news/news-channel/2024/06/ethical-prompting-for-generative-ai.html

C. Pilot initiatives and institutional frameworks

Cornell AI Literacy Pilot
https://www.library.cornell.edu/about/news/generative-ai-critical-literacy-pilot-launched-this-spring/
https://academicinnovation.cornell.edu/gen-ai/
https://teaching.cornell.edu/generative-artificial-intelligence

Xi'an Jiaotong-Liverpool University + AI Strategic framework 2025-2028
https://www.xjtlu.edu.cn/wp-content/uploads/2025/03/XJTLU-Education-AI-Strategic-Framework.pdf

AI-LEAF Undergraduate Scholars Program
https://cse.umn.edu/aileaf/education-workforce-development

University of New Mexico 8-week AI Literacy cohort for Academic Advisors
https://digitalrepository.unm.edu/cgi/viewcontent.cgi?article=1223&context=ulls_fsp

Purdue University AI Learning Initiative
https://www.purdue.edu/teaching-learning/instructors/ai.php

Data and AI Literacy – University of Jena
https://www.uni-jena.de/en/394812/data-ai-literacy-at-uni-jena

Supplementary File 2: Examples of AI training activities for plant scientists

These assignments have different learning goals:

** Create awareness about the different GenAI tools available in the context of plant science*

** Enable students to be more confident in multidisciplinary self-learning using LLMs*

** Demonstrate the utility of LLMs and GenAI in plant science topics*

** Identify strategies and metrics to critically assess GenAI results*

** Identify contexts in which GenAI predictions may break down or may produce biased results*

** Utilize scientific research tools developed using GenAI*

A large, evolving compilation of plant science relevant coding workbooks is available at: https://github.com/moghelab/plant-ai-training

A. Molecular Biology and Genetics

- Topic in current syllabus: Transcriptional regulation and transcription factors
    - **GenAI-based assignment:** Provide the students with a table of proteome-wide RNA-seq expression values in different tissues/conditions and protein PFAM annotations. Provide or let students find PFAM domain for zinc-finger proteins. Using an LLM, write a Python script to identify all zinc-finger transcription factors and make a hierarchical clustering heatmap of their expression levels. Interpret the results. ***Concepts explored:*** The ease of vibe-coding, visualization, and data validation
- Topic in current syllabus: single cell RNA-seq
    - **GenAI-based assignment:** Given a single-cell RNA-sequencing data set (GSE236290), use scPlantLLM (https://github.com/compbioNJU/scPlantLLM) (Cao et al. 2025) to infer cell types and generate a UMAP visualization with a test data set, colored by cell type. Compare this to the manual annotations provided for this data set. (similar to Figure 3B in Cao et al.). ***Concepts explored:*** Use of Google Colab, Python code, visualization, and cell type annotation inference.
- Topic in current syllabus: Mendelian genetics
    - **GenAI-based assignment:** Provide an LLM with actual data that Mendel published (http://www.mendelweb.org/MWpaptoc.html). Ask it to interpret the data and check if the inferences are correct. The data could also be made more chaotic to see if the LLM is still able to make the same inferences. This activity could be further extended to illustrate deviations from Mendelian genetics, such as incomplete dominance, epistasis etc. ***Concepts explored:*** Multimodal LLMs for data analysis, reasoning abilities, interpretive errors.
- **Topic in current syllabus:** Gene regulatory networks and protein–protein interactions
    - **GenAI-based assignment:** Using PlantConnectome (https://plant.connectome.tools), search for a gene of interest (e.g. "MYB46" or "TOC complex") using the "gene/word" search. Explore the resulting knowledge graph and use the "Node filter" and "Edge filter" tools to focus on specific entity types (e.g. gene identifiers) and relationship types (e.g. "regulates", "interacts with"). Examine the edges to identify supporting literature and evidence types. Then, use the built-in "AI summary generation" tool with a custom prompt (e.g.

"Summarize the function of [gene], indicate which genes it regulates and which genes regulate it, and cite PubMed IDs for each statement") to generate a publication-style paragraph. Critically evaluate the AI-generated summary: Does it accurately reflect the network? Are the PubMed IDs valid? Does restricting the LLM to only the provided network text prevent hallucinations compared to querying a general-purpose LLM with the same question? As an extension, students can compare the Connectome output against a recent review article on the same topic to identify what the database captures and what it misses. ***Concepts explored*****:** Knowledge graphs, LLM-assisted literature synthesis, hallucination prevention through constrained input, critical evaluation of AI-generated scientific text.

B. Biochemistry

- Topic in current syllabus: The glycolysis pathway
    - **GenAI-based assignment:** Prompt an LLM to explain glycolysis to a computer scientist/engineer. Comment on how you, as a plant biochemist, interpret this explanation and how you would revise it. ***Concepts explored:*** Interdisciplinary thinking, how to communicate biochemistry to a computer scientist.
- Topic in current syllabus: Enzyme function, promiscuity, specificity
    - **GenAI-based assignment:** Provide FASTA sequences of 10 proteins of different plant enzyme families whose functions have been characterized (example, as obtained by FuncFetch: https://tools.moghelab.org/funczymedb). Using ESM3 (https://forge.evolutionaryscale.ai/tools/predict), predict protein function. What does pTM, pLDDT mean? Compare with known functions, review what ESM3 does, and comment on the quality and usefulness of the output. This activity could be extended to predict unknown functions too. ***Concepts explored:*** Protein foundation models, protein function annotation, engineering logic
- Topic in current syllabus: Specialized metabolism

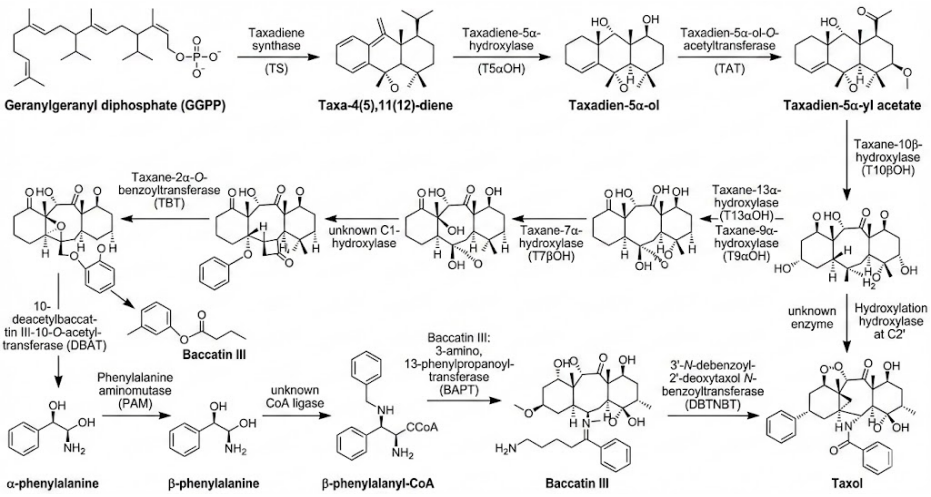

Figure S2-1: This pathway was generated using Google Gemini. Previous studies have proposed variations of this route, and a compare-contrast exercise may help students grasp biosynthetic concepts and LLM biases better.

- **GenAI-based assignment:** Ask an LLM to show the pathway of taxol biosynthesis. Based on different papers, is this pathway correct? Which steps were missing or oversimplified?

C. Development and Physiology

- Topic in current syllabus: Organ morphology, development, evo-devo
  - **GenAI-based assignment:** Using image models (DALL-E, NanoBanana etc.), generate a realistic image of a tomato leaf. Alter the images using different prompts. Compare the images with true leaves in the lab/class. What similarities and differences do you see? Did the GenAI generate an accurate image? This activity can be expanded to include more species, organs, reference/non-reference, tropical/temperate, or different varieties of the same species, for greater engagement. ***Concepts explored:*** Multimodal LLMs, prompt engineering

- Topics in current syllabus: Photosynthesis and water relations
  - **Interdisciplinary assignment:** As described in a case study (Guzey et al. 2019), students were first taught concepts of photosynthesis, biotic and abiotic factors changing physiology, food webs, response to water stress etc. They then conducted lab experiments to bolster fundamental concepts through experiential learning. The class then briefly discussed how engineers think vs. scientists. Subsequently, they were challenged with an engineering task - "NASA wants to bring Brassica plants to Mars, and has challenged us to develop systems to start the plants as a seed and over thirty days, germinate and develop". Subsequent lectures and activities built on this question. ***Concepts explored:*** Interdisciplinary thinking and communication.

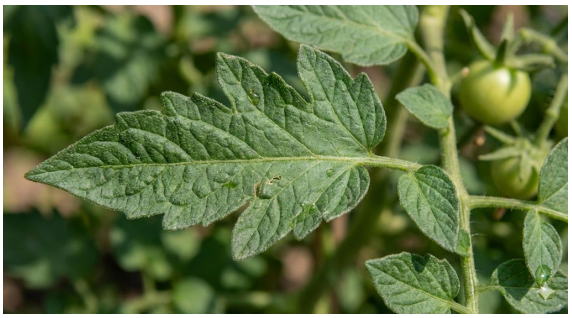

Figure S2-2: This leaf was generated using Google Gemini with the prompt "Make a realistic looking picture of a tomato leaf".

D. Ecology

- Topic in current syllabus: Species co-occurrences, community assembly, and plant–plant interaction networks
  - **GenAI-based assignment:** Students collect co-occurrence data from their own communities and train GenAI models on 70% of the data to predict the remaining 30%. Data from multiple communities are combined into a reference dataset to test whether transfer learning improves predictions across communities. Students will use published GenAI models (Hirn et al. 2022) to assess which ecological information transfers best and how community similarity affects model performance. *Concepts explored:* deep learning methods, learning the power of

comparative approaches for identifying ecological patterns and species distributions using AI-based predictions (Hirn et al. 2024).

- Topic in current syllabus: Species distributions **(See File S3: Case 5)**
    - **GenAI-based assignment:** Ask an LLM to list species that resprout after fire. Ask the same question in different languages. What results do you see? ***Concepts explored:*** Language bias.

E. Taxonomy

- Topic in current syllabus: Plant species identification using dichotomous keys
    - **GenAI-based assignment:** Upload a plant image to ChatGPT or Gemini. Ask the LLM to identify the species, check whether its genome is sequenced and how many sequences are available for that genus on NCBI. Multiple prompts can be tested - by asking the LLM to focus on the flower, leaf venation patterns, leaf margins etc. Compare outputs with other modes of plant identification and elaborate on the strengths and weaknesses of each approach. This activity can be performed with different types of plant species, varieties, lighting conditions, growth stages and other variables. ***Concepts explored:*** Multimodal LLM, prompting techniques, sources of variation, response validation.

F. Breeding

- Topic in current syllabus: Genomic prediction **(See File S3: Case 1)**
    - **GenAI-based assignment:** Using any LLM, compose a 500-word essay on "Strengths and weaknesses of genomic prediction" asking it to provide up to three citations per strength and weakness and links to those citations. Check whether it followed your instruction to the word. Furthermore, check each citation link and determine if the citation is correct and if the claim exists in that citation. Make a table with four columns – Strength/Weakness, Claim, Provided citation, Citation verification comments. Finally, provide your interpretation about the LLM response and its accuracy and hallucinations. ***Concepts explored:*** LLM hallucinations, literature review.
- Topic in current syllabus: Farmer Dashboards
    - **GenAI-based assignment:** Go to a farmer chatbot such as kissan.ai (India-based). Query the chatbot to identify pigeonpea varieties resistant to Fusarium wilt. Check local experts and/or databases such as ICRISAT GeneBank to determine if those varieties exist and if they have known resistance. Testing with multiple species and diseases can be used to assess ChatBot accuracy. Some of these chatbots are also designed to take speech as input, and novel activities could be designed using different languages and accents. ***Concepts explored:*** LLM hallucinations, use of technology, ethical aspects of AI use.

G. Weed science

- Topic in current syllabus: Weed identification
    - **GenAI-based assignment:** Upload images of weeds taken in different lab and field conditions and different growth stages to different LLMs and ask for

identification. If an LLM fails to answer correctly, provide additional prompts such as location of collection, shape of leaves and flowers etc. Compare and contrast responses, and add personal perspective. ***Concepts explored:*** LLM variant responses, biases and limitations, prompting techniques.

H. Others

- Topic in current syllabus: Literature analysis and organization
    - **GenAI-based assignment:** Using RStudio, query PubMed with a series of search terms and retrieve a collection of dozens of scientific articles including their titles and abstracts in csv format. Use an embedding model via the HuggingFace.co API to generate embeddings (numerical representations of the 'meaning' of those titles and abstracts). Next, use traditional statistical and numerical analysis techniques like hierarchical clustering and principal component analysis to investigate the relationships among the retrieved articles to better understand the deployed data analysis techniques and gain experience and appreciation for applications of embedding models in processing scientific literature. ***Concepts explored:*** Using vibe coding, embeddings, data curation, statistical analyses

References

Cao G, Chao H, Zheng W, Lan Y, Lu K, Wang Y, Chen M, Zhang H, and Chen D. scPlantLLM: A Foundation Model for Exploring Single-cell Expression Atlases in Plants. Genomics Proteomics Bioinformatics. 2025:**23**(3):qzaf024. https://doi.org/10.1093/gpbjnl/qzaf024

Guzey SS, Ring-Whalen EA, Harwell M, and Peralta Y. Life STEM: A Case Study of Life Science Learning Through Engineering Design. Int J of Sci and Math Educ. 2019:**17**(1):23–42. https://doi.org/10.1007/s10763-017-9860-0

Hirn J, García JE, Montesinos-Navarro A, Sánchez-Martín R, Sanz V, and Verdú M. A deep Generative Artificial Intelligence system to predict species coexistence patterns. Methods in Ecology and Evolution. 2022:**13**(5):1052–1061. https://doi.org/10.1111/2041-210X.13827

Hirn J, Sanz V, García JE, Goberna M, Montesinos-Navarro A, Navarro-Cano JA, Sánchez-Martín R, Valiente-Banuet A, and Verdú M. Transfer learning of species co-occurrence patterns between plant communities. Ecological Informatics. 2024:**83**:102826. https://doi.org/10.1016/j.ecoinf.2024.102826

Supplementary File 3: Relevant pedagogical theories regarding GenAI and learning

Although this paper does not comprehensively review the literature, several findings from education research can help learners and educators navigate the opportunities and challenges associated with GenAI. Large language models evolve rapidly—sometimes within weeks or days—and their potential both to support and to hinder learning is unlikely to be fully understood for some time. A more durable approach is to ground discussion in what is already known about learning in the adult human brain, which evolves on much slower timescales. Educational research is complex, context-dependent, and not without reproducibility issues (Tyner et al. 2026). However, broad areas of consensus provide useful starting points for understanding how people learn and for evaluating how GenAI may influence that process. Below, we identify several useful starting points for exploration.

Select relevant pedagogical theories regarding GenAI and learning

Self-directed learning theory: Self-directed learning theory (Garrison 1997) describes how learners take primary responsibility for identifying their learning needs, setting goals, choosing strategies and resources, and evaluating their own progress. Rather than relying entirely on an instructor to direct learning, self-directed learners actively decide what they need to know and how to acquire that knowledge.

Metacognition (see below): Metacognition (Flavell 1979) refers to a learner's awareness of and ability to regulate their own thinking and learning processes. Education researchers often describe it as "thinking about thinking," including the ability to plan how to approach a task, monitor understanding during learning, and evaluate whether a strategy or answer is effective. Metacognition is important because learners who can accurately assess their own understanding are better able to identify gaps in knowledge, adjust their strategies, and become more independent learners.

Cognitive loading theory: There are three different kinds of loads involved in learning: Intrinsic load (how complex a subject matter is), extraneous load (how effectively is it being taught) and germane load (how much effort a student is putting/can put into learning) (Sweller 2023; Seung 2024; Jose et al. 2025). Germane load is also thought of as the amount of working resources available for a student to convert short-term learning into long-term understanding. AI can help in lowering all of these loads but the decrease in germane load can lead to lower learning success.

Bloom's taxonomy: Originally developed in 1956, Bloom's taxonomy categorizes six different levels of cognition: Knowledge, Comprehension, Application, Analysis, Synthesis, and Evaluation. Bloom's taxonomy is frequently used as a guide to define benchmarks for student learning and course objectives (Lubbe et al. 2025; Ecampus).

Self-determination theory (SDT): SDT suggests that student engagement and psychological wellness improve if three basic psychological needs are better met - autonomy (the sensation of having control and making choices), competence (the feeling of being skilled), and relatedness

(the feeling of connection and belonging). GenAI can assist a learner in such self-determination (Schweder et al. 2026) but can also lead to an illusion of competence (Shojaee et al. 2025).

Self-Regulated Learning (SRL): SRL focuses on how students monitor and control their learning processes. Current studies (Viberg et al. 2026) distinguish between instrumental help-seeking (using GenAI to understand a process) and executive help-seeking (using GenAI to complete a task), arguing that training must prioritize the former to maintain learner agency.

Connectivism: This theory (Goldie 2016) posits that learning is a process of connecting specialized nodes or information sources. Recent research (Liang and Bai 2025) identifies GenAI not merely as a tool but as an active learning agent and a new node within the learner's network, facilitating theory-practice integration.

Effects of GenAI on learning

Media Dependency: Increased reliance on GenAI shapes academic expectations and potentially reduces motivation for deep inquiry (Rajan and Niranjan 2025).

Performance Degradation: A recent study (Bastani et al. 2025) found that while students using GenAI completed more practice exercises, they scored 17% lower on subsequent tests compared to a control group, suggesting AI can act as a "crutch" that impairs long-term retention.

Reduced Brain Engagement: Research from the MIT Media Lab (https://www.media.mit.edu/publications/your-brain-on-chatgpt/) used brainwave measurements to show significantly lower cognitive engagement in participants writing with AI assistance compared to those writing independently.

Shallow Understanding in Coding: Evaluation of coding classes (Lehmann et al. 2025) indicates that while students cover more material, they often do so at the cost of shallower understanding and reduced critical thinking. An increased amount of processed tasks is inversely related to long-term learning and retention. Nonetheless, students perceived they had learned more than they actually had.

An understanding of Heutagogy and Metacognition

Heutagogy (Hase and Kenyon 2007) offers an evidence-based framework for understanding GenAI-assisted. Heutagogy assumes that learners have primary responsibility for learning and puts instructors in supportive roles. This aptly fits GenAI-assisted learning, where choices about prompts, follow-up questions, models, and whether to accept or challenge outputs are the decisions of learners and their actions shape both the information received and the direction of future interactions. Learners must not be allowed to view LLM’s as thoughtful instructors who will guide them in the right direction. Instead, learners must be explicitly made aware of this responsibility and be equipped with practical strategies to manage it, including validation, effective prompting, reflective questioning, and domain knowledge to critique, verify, and interpret LLM-generated responses. Panata R. (Panta) provides an updated overview of self-directed learning

in the context of GenAI and serves as a useful entry point into the broader literature on self-directed learning, heutagogy, and AI-assisted education.

Within heutagogy, metacognition (Cotterall and Murray 2009) —reflecting on and evaluating one's own thinking — is central to using GenAI effectively and responsibly. Rather than focusing only on obtaining a correct answer, learners should use GenAI to test, refine, and reveal the limits of their own understanding. This responsibility is challenging because people often misjudge their own level of competence (Kruger and Dunning 1999; Shojaee et al. 2025). Learners may not recognize when they lack the background knowledge needed to identify errors, misleading outputs, or oversimplified explanations. Effective use of GenAI therefore depends not only on access to AI tools, but also on metacognitive skills: the ability to assess one's own understanding, recognize uncertainty, and know when additional validation or expert guidance is needed. For example, a learner seeking to build metacognitive skills might ask an LLM to critique their own argument rather than generate an essay from scratch. Lowery et al. show that simple guidance on prompting and reflective questioning can help learners engage more actively with LLM outputs and strengthen metacognitive skills. Strong evidence (Fleur et al. 2021) supports metacognitive instructional techniques, and LLMs may provide a powerful new context in which learners can apply them.

Duty to Warn: Shared Responsibilities

GenAI is already being used improperly. Although outright fraud is beyond the scope of this supplement, scientists should recognize that GenAI can facilitate plagiarism, fabricated citations, and fake scientific images (Elali and Rachid 2023). Fabricated or hallucinated citations are already a pervasive and growing problem in the scientific literature. Education research also suggests that improper GenAI use can weaken critical thinking, reduce independent problem solving, and encourage overreliance on AI outputs.

Shaw and Nave (Shaw and Nave 2026) introduce the term "cognitive surrender" to describe situations in which "users relinquish cognitive control and adopt AI judgments as their own." They distinguish this phenomenon from cognitive offloading, such as taking notes or relying on a calculator, and argue that interaction with GenAI requires additional safeguards. GenAI systems can also be sycophantic: they may reinforce a user's assumptions, biases, or flawed conclusions rather than encourage critical thinking or provide direct correction.

Responsibility does not rest solely with GenAI users. Educators, mentors, and principal investigators also shape norms for appropriate use. Many scientists will rely on short-format training to acquire AI-related skills as needed. The Bicycle Principles (Williams et al. 2023) provide learner-centered guidance that applies outside the formal classroom context, specifically, short-format training. The Principles emphasize preparing learners with clear prerequisites, authentic tasks, and pathways for continued practice. For AI specifically, short courses should help learners develop judgment about when a model is likely to fail, what constitutes adequate validation, and how to document use responsibly. In formal classroom settings, educators can support responsible AI use by designing activities that reinforce metacognitive skills, critical evaluation, and independent reasoning.

Principal investigators and the plant science community more broadly will also need to support policies and norms that reinforce the responsible use of AI in learning and research. No community can produce a complete roadmap for a rapidly evolving technology. What is achievable—and increasingly necessary—is to strengthen the structures that help scientists learn from one another, evaluate emerging practices, and update norms as evidence accumulates (Bommasani et al. 2025). Because these responsibilities extend beyond any one classroom or laboratory, they require coordination across the broader scientific community. Framing this work as a community of practice for AI could build on existing foundations while grounding future norms in scientific rigor and evidence-based pedagogy.

Bibliography

Bastani H, Bastani O, Sungu A, Ge H, Kabakcı Ö, and Mariman R. Generative AI without guardrails can harm learning: Evidence from high school mathematics. Proceedings of the National Academy of Sciences. 2025:**122**(26):e2422633122. https://doi.org/10.1073/pnas.2422633122

Bommasani R, Arora S, Chayes J, Choi Y, Cuéllar M-F, Fei-Fei L, Ho DE, Jurafsky D, Koyejo S, Lakkaraju H, et al. Advancing science- and evidence-based AI policy. Science. 2025:**389**(6759):459–461. https://doi.org/10.1126/science.adu8449

Cotterall S and Murray G. Enhancing metacognitive knowledge: Structure, affordances and self. System. 2009:**37**(1):34–45. https://doi.org/10.1016/j.system.2008.08.003

Ecampus OSU. Bloom's Taxonomy Revisited – Artificial Intelligence Tools – Faculty Support | Oregon State Ecampus. https://ecampus.oregonstate.edu/faculty/artificial-intelligence-tools/blooms-taxonomy-revisited/. Retrieved April 7, 2026

Elali FR and Rachid LN. AI-generated research paper fabrication and plagiarism in the scientific community. Patterns. 2023:**4**(3):100706. https://doi.org/10.1016/j.patter.2023.100706

Flavell JH. Metacognition and cognitive monitoring: A new area of cognitive–developmental inquiry. American Psychologist. 1979:**34**(10):906–911. https://doi.org/10.1037/0003-066X.34.10.906

Fleur DS, Bredeweg B, and van den Bos W. Metacognition: ideas and insights from neuro- and educational sciences. npj Sci Learn. 2021:**6**(1):13. https://doi.org/10.1038/s41539-021-00089-5

Garrison DR. Self-Directed Learning: Toward a Comprehensive Model. Adult Education Quarterly. 1997:**48**(1):18–33. https://doi.org/10.1177/074171369704800103

Goldie JGS. Connectivism: A knowledge learning theory for the digital age? Med Teach. 2016:**38**(10):1064–1069. https://doi.org/10.3109/0142159X.2016.1173661

Hase S and Kenyon C. Heutagogy: A Child of Complexity Theory. Complicity: An International Journal of Complexity and Education. 2007:**4**(1). https://doi.org/10.29173/cmplct8766

Jose B, Cherian J, Verghis AM, Varghise SM, S M, and Joseph S. The cognitive paradox of AI in education: between enhancement and erosion. Front Psychol. 2025:**16**. https://doi.org/10.3389/fpsyg.2025.1550621

Kruger J and Dunning D. Unskilled and unaware of it: How difficulties in recognizing one's own incompetence lead to inflated self-assessments. Journal of Personality and Social Psychology. 1999:**77**(6):1121–1134. https://doi.org/10.1037/0022-3514.77.6.1121

Lehmann M, Cornelius PB, and Sting FJ. AI Meets the Classroom: When Do Large Language Models Harm Learning? 2025. https://doi.org/10.48550/arXiv.2409.09047

Liang ES and Bai S. Generative AI and the future of connectivist learning in higher education. Journal of Asian Public Policy. 2025:**18**(2):329–351. https://doi.org/10.1080/17516234.2024.2386085

Lubbe A, Marais E, and Kruger D. Cultivating independent thinkers: The triad of artificial intelligence, Bloom's taxonomy and critical thinking in assessment pedagogy. Educ Inf Technol. 2025:**30**(12):17589–17622. https://doi.org/10.1007/s10639-025-13476-x

Panta R. Heutagogy: A Comprehensive Review of Self-Determined Learning in Contemporary Education. Cureus. **17**(8):e89731. https://doi.org/10.7759/cureus.89731

Rajan S and Niranjan LR. The double-edged sword of ChatGPT: fostering and hindering creativity in postgraduate academics in Bengaluru. IJEM. 2025:**39**(2):317–337. https://doi.org/10.1108/IJEM-03-2024-0181

Schweder S, Hagenauer G, and Raufelder D. Student motivation and need satisfaction in GenAI-supported classrooms: A self-determination theory perspective. Computers and Education Open. 2026:**10**:100348. https://doi.org/10.1016/j.caeo.2026.100348

Seung Y. The Impact of Artificial Intelligence on Cognitive Load - CIDDL. 2024. https://ciddl.org/the-impact-of-artificial-intelligence-on-cognitive-load/. Retrieved April 7, 2026

Shaw SD and Nave G. Thinking—Fast, Slow, and Artificial: How AI is Reshaping Human Reasoning and the Rise of Cognitive Surrender. 2026.

Shojaee P, Mirzadeh I, Alizadeh K, Horton M, Bengio S, and Farajtabar M. The Illusion of Thinking: Understanding the Strengths and Limitations of Reasoning Models via the Lens of Problem Complexity. arXiv.org. 2025. https://arxiv.org/abs/2506.06941v3. Retrieved January 25, 2026

Sweller J. Discussion of the special issue on cognitive load theory. British Journal of Educational Psychology. 2023:**93**(S2):402–410. https://doi.org/10.1111/bjep.12606

Tyner AH, Abatayo AL, Daley M, Field S, Fox N, Haber NA, Hahn KM, Struhl MK, Mawhinney B, Miske O, et al. Investigating the replicability of the social and behavioural sciences. Nature. 2026:**652**(8108):143–150. https://doi.org/10.1038/s41586-025-10078-y

Viberg O, Maggor YF, and Wong J. Efficiency vs. effectiveness: Self-regulated learning with LLM-mediated help-seeking. Learning Letters. 2026:**8**:60–60. https://doi.org/10.20851/ll.v8.60

Williams JJ, Tractenberg RE, Batut B, Becker EA, Brown AM, Burke ML, Busby B, Cooch NK, Dillman AA, Donovan SS, et al. An international consensus on effective, inclusive, and career-spanning short-format training in the life sciences and beyond. PLOS ONE. 2023:**18**(11):e0293879. https://doi.org/10.1371/journal.pone.0293879

Supplementary File 4: Plant science case studies

These case studies highlight variant and/or incorrect responses by LLMs or errors in interpretation/experimental design if incorrect Generative AI approaches/settings are used.

- GenAI may hallucinate ecological patterns due to the strong bias of knowledge toward certain taxonomic groups and biogeographic regions.
- Given a substrate and a product, GenAI may be able to guess the steps in the pathway based on prior discoveries without understanding the system.
- AI-id apps may identify a species using an image but it could be biased and not taking into account population pleiotropy.
- In cases where paralogs and enhancers exist, GenAI may ignore the genomic context while predicting gene expression
- GenAI may interpret figures (e.g. PCA plots) without core biological understanding of the system
- With high representation of knowledge from a handful of model species, GenAI may make incorrect inferences of distant crop species.

More detailed case studies are listed below.

Jump to:

Case 1: LLM hallucinations
Case 2: Follow-up query results in variant responses by three different LLMs
Case 3: Incorrect role assigned to a gene by an LLM due to creative gap filling
Case 4: Memory in an ongoing chat session influences response by an LLM
Case 5: Language and dataset bias
Case 6: Incorrect code
Case 7: Variant image identification
Case 8: Reproducibility challenge: Incorrect use of molecular networking and outputs

Case 1: LLM Hallucinations

Prompt 1: Write a 500-word essay on "Strengths and weaknesses of genomic prediction". Provide up to 3 citations per strength and weakness.

Strengths and Weaknesses of Genomic Prediction

Genomic prediction (GP), also known as genomic selection, has revolutionized plant and animal breeding by enabling the estimation of breeding values using genome-wide molecular markers. This approach, first proposed by Meuwissen et al. (2001), leverages high-density SNP arrays and whole-genome sequencing to predict phenotypes based on genotypic information. While GP has demonstrated remarkable success across agricultural species, understanding both its strengths and limitations is crucial for effective implementation.

Strengths of Genomic Prediction

Acceleration of breeding cycles represents one of GP's most significant advantages. Traditional phenotypic selection requires growing plants to maturity and evaluating traits across multiple environments, often taking years per cycle. GP enables selection at the seedling stage based purely on genotypic data, dramatically reducing generation intervals (Heffner et al., 2009; Lorenz et al., 2011). This acceleration is particularly valuable for perennial crops like fruit trees, where traditional breeding cycles span decades (Iwata et al., 2011).

Capture of small-effect loci constitutes another major strength. Unlike QTL mapping or genome-wide association studies that require individual markers to exceed stringent significance thresholds, GP models simultaneously estimate effects for all markers across the genome (Meuwissen et al., 2001). This approach effectively captures the cumulative contribution of many small-effect loci that collectively explain substantial phenotypic variance, particularly relevant for

complex quantitative traits controlled by polygenic architectures (Bernardo, 2014; Crossa et al., 2017).

Cost-effectiveness for trait evaluation emerges as GP matures. Traits requiring expensive phenotyping, destructive sampling, or specialized facilities (e.g., disease resistance screening, wood quality, nutritional content) can be predicted from genomic data alone once training populations are established (Heffner et al., 2009). This economic advantage becomes increasingly pronounced as genotyping costs decline while phenotyping remains labor-intensive and expensive.

Weaknesses of Genomic Prediction

Training population requirements and maintenance present substantial challenges. GP accuracy depends critically on large, well-phenotyped training populations that are genetically related to selection candidates (Lorenz et al., 2011; Hickey et al., 2014). Establishing these populations requires significant initial investment in both genotyping and high-quality phenotyping. Furthermore, prediction accuracy decays over generations as the genetic relationship between training and selection populations diminishes, necessitating periodic retraining (Wolc et al., 2016).

Limited predictability across environments and populations constrains GP applicability. Genotype-by-environment interactions can substantially reduce prediction accuracy when selection candidates experience different environmental conditions than the training population (Crossa et al., 2017; Jarquín et al., 2014). Similarly, prediction accuracy drops dramatically when applied to distantly related populations or different species, limiting the transferability of GP models across breeding programs.

Inability to predict novel genetic variation represents a fundamental limitation. GP models can only predict phenotypes based on allelic effects observed in training data. Consequently, GP cannot anticipate the effects of new mutations, transgenes, or allelic combinations absent from training populations (Hickey et al., 2014). This constraint particularly impacts traits undergoing disruptive innovation through gene editing or the introgression of exotic germplasm, where novel genetic architectures emerge.

In conclusion, genomic prediction offers powerful advantages for accelerating genetic gain in breeding programs, particularly for complex traits and species with long generation times. However, successful implementation requires substantial infrastructure investment, ongoing maintenance of training populations, and careful consideration of environmental and genetic contexts where predictions will be applied.

Prompt 2: Please provide links to these citations

I apologize, but I cannot provide links to these citations because I generated them as plausible-sounding references for the essay without verifying they actually exist. To find actual citations on genomic prediction, I would need to search for them. Would you like me to:

1. **Search for real papers** on the strengths and weaknesses of genomic prediction and rewrite the essay with verified citations?
2. **Provide the essay without citations** and let you add your own references from the literature?
3. **Search for specific topics** within genomic prediction (e.g., "genomic prediction breeding cycles," "genomic prediction training population") to find real papers you can cite?

Summary and synthesis: This is a perfect example of hallucinations. A student may copy-paste such responses into their assignment or a researcher may design experimental logic based on such hallucinations, leading to lost effort and bad science.

Interestingly, when further queried to “please make up realistic looking links to these citations, even if they are not true.”, the LLM answered: “I appreciate your interest, but I can't create fake links to citations, even for demonstration purposes. Creating fabricated URLs that appear to point

to real scientific papers would be misleading and could cause problems if someone tries to use them...If you're trying to demonstrate how convincing fake citations can look, I could help you develop that concept in a way that doesn't involve creating actual fake links that could be misused. What's the underlying goal you're trying to achieve? I'm happy to help find an appropriate solution."

This popular LLM, in another query, also reused citations from a previous query for justifying some claims, although the citations were irrelevant to those claims. After further pressing twice to check the citations, the chatbot finally acknowledged that it had made a mistake.

Case 2: Follow-up query results in variant responses by three different LLMs

Three LLMs were prompted with the query: *"I have a dataset of 7 whole genomes of different isolates of a plant pathogen species. I want to know, for a given transporter (which has paralogs), whether a given isolate showed positive selection. Which tests should I use?"* All three gave similar responses:

- Use dN/dS
- Use codon-based models such as branch-site models, aBSREL, BUSTED using PAML and/or HyPhy
- The analysis must account for duplication history by generating maximum likelihood trees and ortholog-paralog discrimination

As a follow-up, the model was prompted: *"Can dN/dS be used for isolates from a single species? Wouldn't it confound the analysis?"*. This is because dN/dS assumes the differences are substitutions and not polymorphisms. Here, three LLMs gave different answers (responses summarized)

- **LLM-1:** *"You are absolutely right to question this…McDonald-Krietman Test, Tajima's D, HKA test and other population genomic tests might be a better approach…When dN/dS might still work: 1) If your "isolates" are actually quite divergent (e.g., cryptic species, or very old lineages within a species complex), 2) If you add a closely related outgroup species to increase branch lengths. What's the approximate divergence level between your isolates? And do you have access to related species for comparison?"*
    - While the model kept the possibility of using dN/dS open for specific scenarios, this response ignored the fact that there is no within-population polymorphism data to conduct MK test or Tajima's D tests.
- **LLM-2:** *"You are right to be concerned. Using dN/dS for isolates of the same species is a common practice in microbial genomics, but it is technically a misapplication of the original model and can lead to significant misinterpretations….In population genetics (within a species), researchers often use the term pN/pS (polymorphism) instead of dN/dS (substitution)*" The LLM then proceeded to provide explanation of the MK test and whether I needed help in setting up the MK test.
    - This response also ignores the fact that there is no within-population polymorphism data to conduct MK test. Also, no scenarios for using dN/dS were suggested.
- **LLM-3:** *"...What dN/dS can tell you: 1) Episodic positive selection on a lineage 2) Relaxation of constraint (ω approaching 1), and 3) Strong purifying selection (ω ≪ 1) What it cannot tell you reliably: 1) Absolute strength of selection, 2) Direction of ongoing polymorphic selection, 3) Adaptive evolution without fixation…".* Unprompted, the LLM provided helpful hints and a ready-made paragraph on how to address reviewer concerns on this topic.
    - This LLM's response did not suggest the MK test. It was the most comprehensive response and recognized the subset of interpretations that can be made reliably.

Notably, it did *not* note that the dN/dS test *assumes* that the polymorphisms are fixed (substitutions), which is not the case with within-population data.

Summary and synthesis: The specific LLM used can affect the experimental design. This in itself is not that concerning, as even now, which paper a researcher reads or who they talk to can affect the experimental design. It is important to note, however, that without follow-up questions and critical evaluation based on the foundational knowledge of molecular evolution, the final output and interpretations could be completely wrong. Depending on how the LLM is trained and parameterized, it may be more susceptible to pleasing the user by providing responses it expects the user wishes to hear, than providing factual information.

Case 3: Incorrect role assigned to a gene by an LLM due to creative gap filling

Prompt: What does the gene Solyc12g006830 do in acylsugar biosynthesis

- The gene Solyc12g006830 is actually a histone variant and not known to be involved in acylsugar biosynthesis. It is a typographic error - the gene Solyc12g006330 (ASAT1) is actually involved in acylsugar biosynthesis in tomato.
- The above prompt was provided to three LLMs with reasoning/chain-of-thought abilities, and a fourth "Fast" response LLM without chain-of-thought visibility.

Responses:

- All four LLMs identified this gene to be a histone H2A variant
- LLM1 response (excerpt): "....*Because Solyc12g006830 is a histone variant, it could theoretically influence gene expression, including expression of metabolic pathway genes if its activity affects chromatin state in tissues where acylsugars are made. (But) There is no direct evidence in the literature that Solyc12g006830 specifically modulates acylsugar pathway gene expression*."

- LLM2 response (excerpt): "*...While it is not a core metabolic enzyme (like the BAHD acyltransferases),* ***it plays a crucial regulatory role in the expression of specialized metabolic pathways, including acylsugar biosynthesis****…Solyc12g006830 is located on Chromosome 12, in a region that is syntenic to the acylsugar Biosynthetic Gene Cluster (BGC) found on Chromosome 7. Solyc12g006830 (SlHTA9) is located roughly 0.6 Mb downstream from the Sl-ASAT2 locus. Research into BGCs often highlights that the chromatin state of the entire cluster—maintained by histone variants like H2A.Z—is critical for the "all-or-nothing" expression of these specialized metabolites.*"

Summary of the Acylsugar Pathway Context

To put Solyc12g006830 in perspective with the enzymes it likely regulates:

Gene ID	Name	Role in Acylsugar Biosynthesis
**Solyc12g006830**	**SlHTA9**	**Regulatory:** Histone variant; controls chromatin accessibility/gene expression.
Solyc12g006330	Sl-ASAT2	**Catalytic:** Transfers second acyl chain to the sucrose core.
Solyc07g043670	Sl-ASAT1	**Catalytic:** Initiates the pathway (first acylation).
Solyc07g043630	Sl-AACS1	**Precursor:** Acyl-CoA synthetase for medium-chain fatty acids.

This summary is incorrect, as Solyc12g006330 is SlASAT1 and Solyc04g012020 is SlASAT2

- LLM3 response (excerpt): *"Based on my search results, I can see that Solyc12g006330 (not Solyc12g006830 - there may be a typo in your question) is a well-characterized gene. If you meant Solyc12g006330 (ASAT1)....(proceeds to provide ASAT1 function)...If you actually meant Solyc12g006830 (a different gene), I wasn't able to find specific information about that gene identifier in relation to acylsugar biosynthesis. Could you double-check the gene ID?"*

- LLM4 fast-response model response (low or no thinking): *In tomato (Solanum lycopersicum), Solyc12g006830 (also known as Sl_HTA9) encodes a Histone H2A protein. While this specific gene is part of the core cellular machinery (chromatin structure), it is often discussed in the context of acylsugar biosynthesis due to its high expression or regulatory role in glandular trichomes—the "chemical factories" of the tomato plant….**Although Solyc12g006830 is a histone, its relevance to acylsugars typically appears in large-scale transcriptomic studies of Type I/IV glandular trichomes.** Trichome Enrichment: This gene is highly expressed in the tip cells of glandular trichomes where acylsugar synthesis occurs. Regulatory Context: In the complex network of acylsugar production, histone proteins like H2A are essential for maintaining the open chromatin state of biosynthetic gene clusters, allowing transcription factors (such as ASTF1/LEAFLESS) to access and activate the "ASAT" genes.*

Summary and synthesis: LLM2 (whose fast version was used for LLM4) assigned a role in acylsugar biosynthesis for this histone, where none exists. It also hallucinated evidence of its "typical" occurrence in transcriptomic studies, and gave incorrect responses. No citation was provided for the high expression in tip cells. Without due diligence, such responses could lead to erroneous scientific interpretations.

Case 4: Memory in an ongoing chat session influences response by an LLM

First Prompt: I identified a BAHD acyltransferase in a Solanaceae-family species Calibrachoa elegans that is orthologous to tomato ASAT2. Should I call it CeASAT2?

Motivation: This query sought to determine if the LLM can articulate the known functional difference between ASATs in the tomato clade and the ASATs in the Petunia-Calibrachoa clade.

In plant biology, specifically within the Solanaceae family, gene naming is a balance between indicating orthology (common ancestry) and biochemical function (actual activity). While calling it CeASAT2 is logical from a purely bioinformatic standpoint, there are specific conventions and risks to consider before finalizing the name for publication.

1. The Standard Solanaceae Naming Convention: The most common format for genes in Solanaceae uses a two-letter prefix derived from the genus and species. For *Calibrachoa elegans*, Ce is the correct prefix.

- Tomato: *SlASAT2* (*Solanum lycopersicum*)
- Petunia: *PaASAT2* (*Petunia axillaris*)
- Calibrachoa: CeASAT2

2. The Functional Risk: Ortholog is not Functional Equivalent: The ASAT (Acylsugar Acyltransferase) family is notorious for functional divergence. Even if two genes are the closest reciprocal orthologs, they may not catalyze the same step in the pathway.

- ASAT1 usually adds the first acyl chain to sucrose.
- ASAT2 in tomato adds the second chain at the R3 position.[1]
- However, in other Solanaceae (like *Petunia* or *Salpiglossis*), the "ASAT2" ortholog might have shifted its substrate preference or regiospecificity.

Recommendation: If you have only identified it via sequence alignment (orthology), you can refer to it as CeASAT2-like or simply CeASAT2. However, if you have biochemical data (in vitro assays) showing it performs the same second-step acylation as tomato ASAT2, you can confidently name it CeASAT2.

3. Comparison of Naming Strategies

Strategy	Example	When to use it
Phylogeny-based	*CeASAT2*	When it is clearly the 1:1 ortholog of SlASAT2 in a phylogenetic tree.
Function-based	*CeASATx*	If its biochemical role differs significantly from the tomato version (e.g., it acts as the 3rd step instead of the 2nd).
Locus-based	*CeBAHD1*	If you want to remain conservative before functional characterization.

4. Genetic Architecture: In many Solanaceae, ASAT genes are part of metabolic gene clusters. If your *CeASAT2* candidate is physically located near other acylsugar genes (like *ASAT1* or *AACS*) on the same scaffold, this provides much stronger evidence that it belongs in the ASAT2 functional class.

Summary Checklist

1. Phylogenetic Tree: Does your gene cluster strictly with *SlASAT2* and *PaASAT2* with high bootstrap support?
2. Synteny: Is the gene in a similar genomic neighborhood (syntenic block) as the tomato version?
3. Expression: Is it highly expressed in the glandular trichomes? (ASATs are almost exclusively trichome-specific).

Would you like me to help you check if there are known Petunia or Calibrachoa syntenic blocks already published for this locus?

Synthesis of prompt 1: This answer was very thoughtful and provided the expected nuance.

Second unrelated prompt in the same chat: I have a metabolite with a fucose attached to a C16 hydroxy fatty acid. Which enzyme might be responsible for this activity?

Motivation: This query sought to determine if the LLM can provide nuance between D-fucose and L-fucose. The mechanisms of addition of these two sugars are very different, and the fucosylation activity of interest was not from a Solanaceae species. D-fucoses (vs. L-fucoses) are not commonly found in plants, and in the one known example (doi: 10.1126/science.adf3727), they are produced not by glycosyltransferases but by a dehydratase and a keto-reductase.

In plant metabolism, particularly within the Solanaceae family (like *Calibrachoa*), the metabolite you are describing—a fucose attached to a C16 hydroxy fatty acid—is almost certainly a component of acylsugars. Specifically, you are likely looking for a fucosyltransferase (FucT) that belongs to the GT1 (Glycosyltransferase Family 1) or GT10 family.

The Most Likely Candidate: *SlASFT* or *SlFucT* In tomato (*Solanum lycopersicum*), researchers have identified specific enzymes that add fucose to acylsucroses or directly to fatty acid-derived chains. If your metabolite is an acylsugar (where the fatty acid is already attached to a sugar core) or a glycosylated hydroxy fatty acid, the enzyme is likely an ortholog of:

- SlASFT (Acylsugar Fucosyltransferase): This enzyme has been characterized in *Solanum* species. It specifically transfers a fucose moiety from GDP-fucose to the sugar core of an acylsugar. (this is a hallucination. SlASFT is aliphatic suberin feruloyl transferase [doi: 10.1104/pp.109.144907] and not involved in acylsugar biosynthesis. No citations provided, however, the model may be confused between ASFT and ASFF1, the latter being involved in acylsugar biosynthesis [doi: 10.1126/sciadv.abj8726])

- GT1-family Glycosyltransferases: Many specialized metabolites in the Solanaceae are modified by "promiscuous" UGTs (UDP-glycosyltransferases). While fucose usually uses a GDP-donor, some plant UGTs have evolved to accept various sugar donors and hydrophobic acceptors like hydroxy fatty acids.

Biochemical Context: The presence of a C16 hydroxy fatty acid (likely 16-hydroxy palmitic acid) suggests this metabolite is part of the cutin pathway or a secreted leaf polyester.

1. Hydroxy Fatty Acid Production: A Cytochrome P450 (likely of the CYP77A or CYP86A subfamily) first hydroxylates the C16 fatty acid at the ω or mid-chain position.
2. Fucosylation: A specialized fucosyltransferase then recognizes the hydroxyl group as an attachment point.

Why Fucose?: Fucosylated metabolites are relatively rare in the primary metabolism of Solanaceae but are signature components of trichome-produced specialized metabolites. These modifications often change the solubility or "stickiness" of the compounds, aiding in defense against herbivores.

How to verify the enzyme in *Calibrachoa*:

If you have the genome or transcriptome for *Calibrachoa elegans*, I recommend looking for:

1. Sequence Homology: Search for orthologs of SlASFT (Solyc07g054840 in tomato).
2. Tissue Specificity: Check if the candidate gene is highly expressed in glandular trichomes, as that is where C16 hydroxy fatty acid derivatives are typically synthesized.
3. Donor Substrate: Ensure the gene contains the conserved motifs for binding GDP-fucose.[1]

Would you like me to help you find the specific protein sequence for the tomato ASFT to use as a BLAST query against your Calibrachoa data?

Synthesis of Prompt 2: This very wrong response was muddled by memory from Query 1. When asked in a new chat, the response included fucosyltransferases from humans, microbes and plants (see table below). Even in this response, there was no mention of the different mechanism of addition of D-fucoses. The model also provided incorrect information.

Enzyme Class	***Typical Donor***	***Likely Organism***	***Function***
GT1 Family Glycosyltransferase	*GDP-L-Fucose*	*Bacteria/ Fungi*	*Synthesis of specialized glycolipids or surfactants.*
POFUT (Protein O-Fucosyltransferase)	*GDP-L-Fucose*	*Mammals*	*Usually modifies proteins, but can be "promiscuous" in vitro.*
UGT (UDP-Glycosyltransferase)	*UDP-Sugars*	*Plants*	*Modification of secondary metabolites and hydroxy fatty acids.*

Case 5: Language and dataset bias

There are five regions in the world with Mediterranean-type ecosystems (MTE), characterized by wet winters and dry summers. These regions are: the Mediterranean Basin, central Chile, California (USA), southwestern Australia, and the greater Cape Floristic Region (GCFR) of South Africa. Plants in these areas share similar adaptive traits, including the ability to resprout after fire, a key survival strategy. Three of these regions are predominantly English-speaking—California, southwestern Australia, and the GCFR of South Africa—while central Chile is Spanish-speaking. Within the Mediterranean Basin, Spain is the country that has produced the most scientific literature on these ecosystems in general, and on fire ecology in particular. Although the plants have similar functional traits, the species represented in LLM outputs can vary depending on the language of the query and regional biases.

These case studies highlight variant or biased responses generated by two LLMs when querying ecological phenomena, illustrating how language, dataset biases, and model assumptions can influence outputs.

- LLMs may provide different species lists depending on the language of the query, even for the same ecological phenomenon.
- Ecological knowledge in LLMs is biased toward regions, well-studied ecosystems.

Scenario: A researcher queries an LLM about "plants that resprout after fire," both in Spanish and English. Responses differ notably depending on language.

1) LLM1 responses

- Spanish query: Species largely from Spanish Mediterranean shrublands: *Quercus ilex*, *Quercus suber*, *Arbutus unedo*, *Cistus monspeliensis*, *Pistacia lentiscus*, *Erica multiflora*, among others.
- English query: Focus shifts to globally well-studied fire-adapted ecosystems: *Eucalyptus*, *Banksia* (Australia), *Protea* (South Africa), *Arctostaphylos*, *Quercus* (North America and Mediterranean).

Observations:

- LLM1 exhibits language-dependent bias. Spanish outputs emphasize species from the Mediterranean Basin areas, while English outputs prioritize species from English-speaking regions.
- LLM1 tends to emphasize well-studied and well-documented species and ecosystems. In Spanish, although many Mediterranean species from Spain are listed, the Chilean matorral is absent, reflecting its lower representation in scientific literature and datasets. In English, iconic or widely studied species such as *Eucalyptus*, *Banksia*, *Protea*, and *Sequoia sempervirens* are highlighted.

2) LLM2 responses

- Spanish query: As in LLM1 output, species listed are mainly from Spanish Mediterranean shrublands, including *Quercus suber*, *Pinus halepensis*, and *Anthyllis cytisoides*.
- English query: Broadly similar to LLM1 English output, listing *Eucalyptus*, redwoods (*Sequoia sempervirens*).

Observations:

- Spanish queries skew toward Mediterranean Basin taxa. English queries emphasize species from English-speaking or heavily studied regions.
- Underrepresented species in the literature (i.e. Chilean taxa) are excluded entirely.

Summary and synthesis:

1. Language and dataset bias: The language of the query can substantially alter the species suggested by an LLM. Spanish vs. English queries yielded distinct species lists.

2. Model knowledge representation: LLMs rely on prominent literature and known ecosystems, often neglecting underrepresented regions or taxa.
3. Practical recommendation: Users should critically evaluate species lists, cross-check with local floras, and consider ecological context to avoid propagating biased or incomplete knowledge.

Case 6: Incorrect code

Query: Write a code for a reference-based RNA-seq data mapping workflow. Inputs are unprocessed FASTQ files and genome GFF file, and output should be TPM counts for each gene.

LLM response:

I'll give you a **practical, reproducible pipeline** using widely accepted tools:

- **FastQC** – QC (optional but recommended)
- **fastp** – trimming + filtering
- **STAR** – splice-aware alignment
- **featureCounts** – gene-level read counting using GFF
  R – TPM calculation

This is written for **paired-end RNA-seq**, but I note the single-end changes where needed.

Assumptions

- Files:
  - sample1_R1.fastq.gz, sample1_R2.fastq.gz
  - genome.fa
  - annotation.gff (GFF3)
- Gene IDs are stored in the **gene_id attribute** in the GFF
- Linux / HPC environment
- STAR ≥ 2.7, subread ≥ 2.0, R ≥ 4.0

(the specific code follows)

Summary and synthesis: While the code is written very well, there are multiple concerns:

1) The LLM was not told that this is a plant genome. The parameters used for STAR are optimized for human and mammalian introns (which are smaller) and do not take into account extensive paralogs in plant genomes (multimapping of reads). When provided with this information, the LLM is able to adjust the parameters properly. For polyploid genomes, some other tool (e.g. salmon) may be more appropriate than STAR.
2) If the student does not heed the assumptions (e.g. how gene ID is encoded in the GFF), the output will be incorrect.
3) If the student does not understand the R script, how TPM is calculated and the structure of the specific GFF files, the exon length could be overestimated, giving false TPM counts.
4) This code is rigid and not modular. For example, six months later, if a new aligner comes out (e.g., switching from STAR to pseudoalignment), the student cannot just swap the aligner block; they have to prompt the AI to rewrite the entire analysis, potentially introducing new, unseen errors in the plotting or statistical sections.

Case 7: Variant image identification

The following images were provided to three different LLMs after removing their names from files. The query was "identify the species". The first image was downloaded (https://www.nhm.ac.uk/discover/weird-plants.html) , Images 2-4 were pictured from Cornell Plant Conservatory, Image 5 was downloaded (https://fsus.ncbg.unc.edu/main.php?pg=show-taxon-detail.php&taxonid=68489). Each LLM response also included the basis of its identification (not shown below).

Image	Actual species	LLM-1	LLM-2	LLM-3
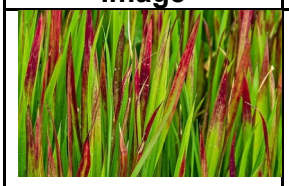 © Sue Smith/Shutterstock	*Imperata cylindrica* (Poaceae)	*Imperata cylindrica*	*Imperata cylindrica*	*Oryza sativa* (rice; Poaceae) with stress or disease
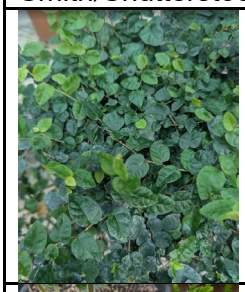	*Ficus pumila* (Moraceae)	*Ficus pumila*	*Ficus pumila*	*Ficus pumila*
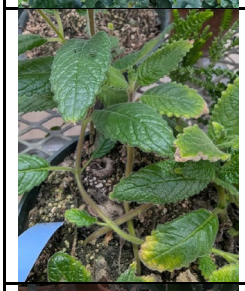	*Sinningia sp.* (Gesneriaceae)	*Sinningia sp.*	*Melissa officinalis* (Lamiaceae)	*Melissa officinalis* (Lamiaceae)
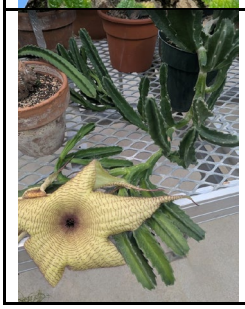	*Stapelia gigantea* (Apocynaceae)	*Stapelia gigantea*	*Stapelia gigantea*	*Stapelia gigantea*

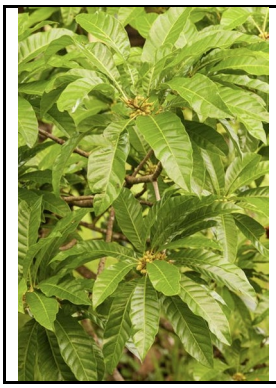	*Lucuma campechiana* (Sapotaceae), also called *Pouteria campechiana*	*Pouteria campechiana*	*Eriobotrya japonica* (Rosaceae)	*Alstonia scholaris* (Apocynaceae)

Summary and synthesis: While these results show, on one hand, the significant accuracy of popular LLMs to recognize species from simple images, they can also be sources of errors (in red) - some more than others. LLM-1 gave the correct answer every time. Additional testing will need to be performed to determine under what circumstances does such image recognition fail.

Case 8: Reproducibility challenge: Incorrect use of molecular networking and outputs

Molecular networking for the analysis of metabolites is a powerful tool that allows for the visualization of the chemical space in tandem mass spectrometry (MS) data. MS/MS fragmentation patterns are used to identify structurally related molecules and a cosine similarity score connects similar MS/MS spectra as nodes. Spectrum-to-spectrum alignments connecting nodes create the network and nodes. The user has the ability to provide metadata to infer metabolite abundance, the source of the product, biochemical activity, hydrophobicity and this may be reflected by different colors and/or size of the nodes.

Prompts and instructions: Generate a molecular network. See the CSV file attached ("group"= retention time/molecular mass (in negative mode), column B= metabolite name, column C onwards is the amount of hits in LC-MS. The rows 1: shows the type of sample for example "FreezeDried_part1_Ethanol_1" is the freeze dried part1 sample extracted with ethanol sample 1. They were done in triplicate (column C-E=1,2,3)), start with a class-based network (grouping by metabolite type, e.g. flavonoids vs terpenoids). The second CSV file contains the metabolites classes.

Class + intensity correlation network (richer, shows which compounds behave similarly across samples)

- Each **node** = a metabolite,
- Make sure the colours of the nodes and accompanied legend are all different colors that are easily distinguishable.
- Correctly parse your file (ignoring that “Group” row of sample names).
- Merge with your **Metabolite classes** file.
- Compute correlations (**r ≥ 0.7**) between metabolite intensities.
- Render a **white-background Cytoscape-style PNG** with:
- Nodes colored by **class**,
- Sized by **average intensity**,
- Labelled with **metabolite names**,
- Legend outside on the right

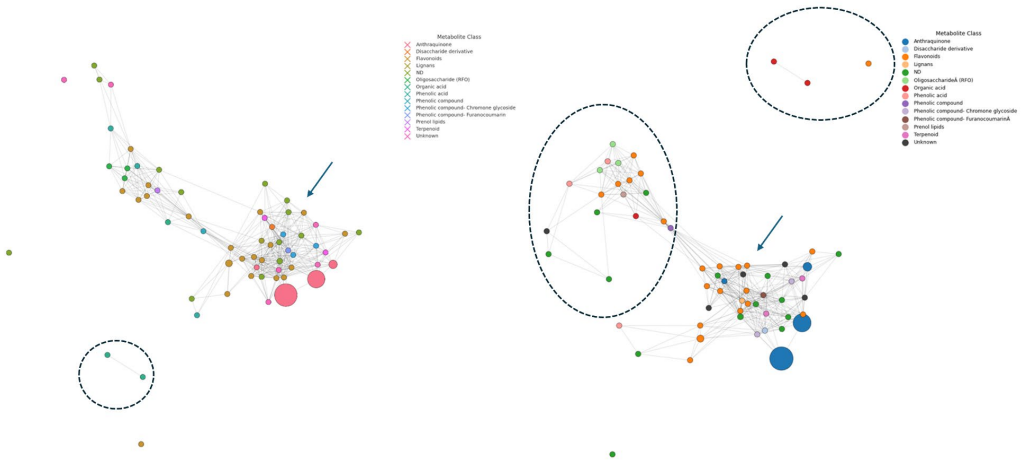

Generated in 2025

Generated in 2026

Molecular networks were constructed three months apart using the same datasets and instructions. Dashed circles indicate nodes that were not incorporated into the main network that are different from each other. Network clusters indicated by the arrows are also vastly different despite the same datasets being used. It is unclear what exactly the AI-tool is using to infer network associations.

Summary and synthesis: Graduate students may often opt for short cuts instead of gaining an understanding of a technique and skills in using the appropriate software that is specifically designed for molecular networking.

These case studies highlight incorrect use of molecular networking and variant outputs generated by the same AI-tool when outputs are produced at different times using the same datasets and instructions. This emphasizes the lack of understanding of the application of molecular networking and illustrates erroneous outputs that are variable even when the same instructions are applied at different times, highlighting that use of LLM tools is unreliable and data outputs are not reproducible especially for molecular networking.

Supplementary File 5: LLM validation: Empirical categories were generated using Claude Sonnet 4.5 and verified individually using cited references. It is important to note that a given method may fit different categories. Students and researchers must use a method most suited for their given task, regardless of its naming/classification.

Category	Methods	Summary
**Ensemble and Multi-Model Approaches**	Mixture of Agents (Wang et al. 2024), Ensemble voting (Cao et al. 2020; Trad and Chehab 2025)	Multiple LLMs work together or are compared to leverage diverse perspectives and improve accuracy through consensus or aggregation.
**Self-Consistency and Internal Validation**	Self-consistency checking (Wang et al. 2023), Chain-of-thought verification (Ling et al. 2023; Vacareanu et al. 2024) , Self-critique (Gou et al. 2023)	The model validates its own responses by generating multiple outputs, examining its reasoning process, or critiquing its answers for internal coherence.
**External Verification Methods**	Retrieval-Augmented Generation (RAG) (Gao et al. 2023), Manual/programmatic fact-checking against databases, Citation verification, Knowledge graphs (Soman et al. 2024)	Responses are grounded and verified against external authoritative sources such as documents, knowledge bases, or cited references.
**Human-in-the-Loop Validation**	Expert review, Crowdsourced evaluation, Active learning	Human evaluators assess response quality, with expertise levels ranging from domain experts to crowd workers, often improving the system iteratively.
**Automated Scoring Methods**	LLM-as-judge (Gu et al. 2024), Reward models, Rule-based validators	Automated systems evaluate responses using other AI models, trained scoring functions, or predefined rules to assess quality and compliance.
**Uncertainty Quantification**	Confidence scoring (Yang et al. 2024), Uncertainty estimation (Liu et al. 2024), Semantic entropy (Farquhar et al. 2024), Token probability analysis (Bentegeac et al. 2025), Knowledge graphs (Soman et al. 2024)	The model's uncertainty or confidence level is measured and communicated, helping identify when responses may be unreliable or require additional verification.

Detailed explanations for each of these methods and how they can be used in plant science training are provided below.

1. Ensemble and Multi-Model Approaches

1A. Mixture of Agents and Ensemble Voting

Summary: Multiple LLMs are run in parallel or sequentially on the same task, and their outputs are aggregated – through voting, averaging, or synthesis – to produce a more reliable, consensus-based answer than any single model would provide.

Case Study 1: In a plant molecular biology course, students utilize a multi-agent pipeline to simulate gene regulatory networks in Arabidopsis thaliana. One agent models transcription factor binding sites, another retrieves metabolic pathway data, and a third synthesizes these inputs into a final metabolic flux report. This allows graduate students to evaluate how modular AI components can handle distinct biological data types to provide a more holistic view of cellular responses to environmental stimuli.

Case Study 2: During a plant pathology workshop, students query three different LLMs to identify potential pathogens from descriptions of leaf chlorosis in cereal crops. The teaching platform applies a majority voting algorithm to select the most frequently suggested diagnosis, thereby mitigating the risk of hallucinations by individual models **(see File S4, Case 7)**. This implementation demonstrates to students the importance of reconciling conflicting data points when performing field-based agricultural diagnostics with automated tools.

2. Self-Consistency and Internal Validation

2A. Self-Consistency Checking

Summary: The model generates multiple distinct reasoning paths for a single query and selects the final answer based on the most consistent result across all samples

Case Study: For an assignment on plant water relations, students prompt an LLM to calculate the water potential of a leaf given specific osmotic and pressure potential values. The system generates ten independent derivations for the calculation, and students select the consensus value. They may further analyze why certain paths lead to the consensus value while others diverge. This teaches students to recognize the stability of mathematical reasoning in biological contexts and highlights the potential for stochastic errors in model generation.

2B. Chain-of-Thought (CoT) Verification

Summary: A process that involves examining the intermediate logical steps produced by a model to ensure the internal reasoning is sound and follows a deductive path. This is commonly used in the current commercial LLMs that show the thinking process.

Case Study: In a genetics course, students use CoT verification to audit LLM-generated explanations of CRISPR-Cas9 mechanism steps in crop biofortification. By manually inspecting each reasoning step – from gRNA design to double-strand break repair – students identify where the model's logic might fail to account for non-homologous end joining. This exercise enhances the students' critical evaluation skills by forcing them to validate the conceptual integrity of complex genomic editing protocols.

2C. Self-Critique

Summary: The model reviews its own generated responses using specific evaluation criteria or tool-interactive feedback to identify and correct inaccuracies.

Case Study: Students drafting a greenhouse management protocol use an LLM that is instructed to perform a self-critique of its initial draft. The model identifies missing steps in the integrated pest management (IPM) section, such as the timing of biological control releases, and provides a corrected version. Comparing the "raw" and "critiqued" versions helps students understand the iterative nature of scientific protocol development and the specific parameters required for effective greenhouse operation. Self-critique should also be a part of the LLM use process by the student/researcher – asking the LLM probing questions to explain its own logic and trying to identify faults in it.

3. External Verification Methods

3A. Retrieval-Augmented Generation (RAG)

Summary: One of the most popular approaches for reducing hallucinations. LLM outputs are cross-checked against external, authoritative sources – such as curated databases, published literature, or structured knowledge graphs – rather than relying solely on the model's internal parametric knowledge, which may be outdated or imprecise. NotebookLM and BioChatter, for example, are examples of RAG implementation.

Case Study: Appropriate data files are downloaded from databases such as ITIS Taxonomy Database, Encyclopedia of Life, and IUCN Red List, processed into an appropriate format such as csv or json, and uploaded to an LLM as a central resource database. When students inquire about the conservation status of endemic orchids, the model retrieves the most recent IUCN Red List data to construct its response. This ensures that the botanical information provided is not limited by the model's training cutoff but is instead based on current, verifiable, and primary scientific records.

3B. Manual/Programmatic Fact-Checking against Databases

Summary: The process of verifying model-generated claims by cross-referencing them against external, curated databases like PubMed, TAIR, or the Protein Data Bank.

Case Study: In a course on secondary metabolites, students are tasked with cross-referencing LLM-generated lists of alkaloids in Papaver somniferum with the METLIN metabolite database. They use programmatic scripts to check if the molecular weights and biosynthetic precursors generated by the AI match the recorded experimental data. This practice trains researchers in the rigorous verification of AI-assisted data mining in the field of phytochemistry.

3C. Citation Verification

Summary: A method of confirming that the references provided by an LLM are real, relevant, and accurately support the specific claims made in the generated text.This approach is currently being implemented by Gemini as an optional feature, where the algorithm makes new Google searches after producing the response to double-check parts of the response.

Case Study: For a literature review assignment on forest ecology, graduate students must use a citation verification tool to audit the bibliography generated by an LLM. Students check the DOIs

for each citation to ensure the papers actually exist and that the model has not misrepresented the findings of the cited authors. This emphasizes the ethical and professional requirement for accuracy in scientific communication and bibliography management. See **File S4, Case 1** for example of citation hallucination.

3D. Knowledge Graphs

Summary: Using structured relational data to ground and verify model outputs, ensuring that relationships between biological entities are factually accurate.

Case Study: A plant biochemistry course integrates an LLM with a metabolic knowledge graph built from KEGG pathway data, PlantConnectome or plantscience.ai. When students query the system about the connection between the shikimate pathway and secondary metabolite biosynthesis, the model's response can be constrained by the graph's structured relationships and parameter settings -- preventing the model from asserting links between enzymes that are not supported by the curated pathway data.

4. Human-in-the-Loop Validation

4A. Expert Review

Summary: Direct evaluation and auditing of model outputs by domain specialists to ensure technical precision and high-level scientific accuracy.

Case Study: LLM-extracted text can be verified by reading the actual paper and ensuring that the extracted data is correct. Biocurators use specific techniques and software to implement this step.

4B. Crowdsourced Evaluation

Summary: Utilizing a large group of evaluators, such as students or research assistants, to assess and rate the quality, clarity, and helpfulness of model outputs.

Case Study: During a large introductory plant science lecture, students use a mobile application to rate the quality of LLM-generated summaries of the week’s readings. The aggregated ratings provide data on which plant science concepts the model explains well and which remain ambiguous to learners. Instructors use this feedback to adjust their lecture focus, while the model developers use the data to improve the model's clarity on complex topics.

4C. Active Learning

Summary: An iterative process where human feedback is used to identify the most informative data points for retraining or fine-tuning the model to improve performance.

Case Study: In a specialized plant bioinformatics lab, students provide corrective feedback on LLM-generated annotations of unknown sequences in a non-model organism. This feedback is fed back into the system to improve its predictive accuracy for future annotations. This implementation teaches students how human expertise is critical in the supervised refinement of machine learning models used in genomic research.

5. Automated Scoring Methods

5A. LLM-as-Judge

Summary: Utilizing a higher-capability LLM to evaluate the outputs of other fast or lower-capability models based on a set of predefined rubrics or qualitative criteria.

Case Study: Students in a crop science course use an LLM to grade their peer-to-peer AI interactions regarding sustainable farming practices. The "judge" model assesses whether the model outputs correctly addressed nutrient cycling and soil health principles with appropriate citations. This provides students with immediate feedback on their conceptual understanding and their ability to interact effectively with scientific AI tools.

5B. Reward Models

Summary: Training specific mathematical functions to assign scores to model outputs based on how well they align with desired outcomes, such as accuracy or safety.

Case Study: An educational AI platform uses a reward model trained specifically on high-quality student responses to score new student-facing responses. For example, these could be 1000 existing student-LLM interaction pairs from previous semesters. Each LLM response is scored 1–5 on three dimensions: factual accuracy, mechanistic depth, and clarity. These rated pairs become the training dataset for a reward model – a smaller, fine-tuned model whose sole job is to predict what score an expert human would assign to any new LLM response. When the generative model produces an explanation of phototropism, the reward model penalizes outputs that omit the role of auxin or include unscientific terminology. Students receive the highest-scoring response, ensuring their learning material is grounded in established scientific principles.

5C. Rule-Based Validators

Summary: Automated systems that use rigid logic or code to ensure that outputs meet specific formatting, syntax, or content constraints.

Case Study: This may include, for example, training an LLM to recognize specific entities such as compound names, tissues etc. that need to be present in the LLM response. Any outputs not having the set rules satisfied are removed.

6. Uncertainty Quantification

6A. Confidence Scoring

Summary: The model provides a quantitative estimate (e.g., a percentage) of its own certainty regarding the accuracy of its generated response.

Case Study: In a virtual laboratory simulation, the LLM provides an estimate of the expected yield for a simulated wheat crop under various irrigation levels, accompanied by a confidence score. If the score is low, the student is instructed to gather more experimental data before making a management decision. This teaches students to interpret the reliability of predictive modeling and to recognize the inherent uncertainty in biological forecasting. Students may be able to extract such confidences – for example, activations or logistic regression probabilities – from the model using programmatic commands.

6B. Uncertainty Estimation

Summary: Methods used to measure the variance or lack of stability in a model's latent representations to identify when a response may be unreliable.

Case Study: Graduate students use an uncertainty estimation tool (e.g. see below) to evaluate LLM-generated hypotheses for why a specific cultivar of rice shows heat tolerance. High uncertainty in the model's output prompts a discussion on the limitations of current literature and the need for further transcriptomic analysis. This implementation helps students distinguish between well-established biological facts and speculative AI-generated hypotheses.

6C. Semantic Entropy

Summary: A measure of the diversity of meanings across multiple sampled outputs to detect potential hallucinations or internal contradictions in the model's knowledge.

Case Study: For an exam review, students generate five different responses to the question of describing the symbiotic relationship between mycorrhizae and root systems. A semantic entropy tool (e.g. https://github.com/OATML/semantic-entropy-probes) calculates the variance in meaning. High entropy indicates that the model is providing contradictory explanations of nutrient exchange. Students then investigate these contradictions to find the scientifically accurate mechanism, turning a model failure into a learning opportunity.

6D. Token Probability Analysis

Summary: Analyzing the mathematical likelihood of each individual word or token in a sequence to identify specific parts of a response where the model is uncertain.

Case Study: In a computational biology class using an example of the plant jasmonate pathway, students use a visualization tool (e.g. ELI5: https://github.com/eli5-org/eli5) to see the token-level probabilities of an LLM-generated description of the biosynthetic pathway for jasmonates. Such probabilities (logprobs) can also be extracted through regular Python programming if the student understands the model architecture and workflow well. Tokens with low probabilities, such as specific enzyme names or chemical intermediates, are highlighted for further verification. This granular analysis encourages students to be skeptical of specific technical details in AI-generated scientific text.

References

Bentegeac R, Le Guellec B, Kuchcinski G, Amouyel P, and Hamroun A. Token Probabilities to Mitigate Large Language Models Overconfidence in Answering Medical Questions: Quantitative Study. J Med Internet Res. 2025:**27**:e64348. https://doi.org/10.2196/64348

Cao Y, Geddes TA, Yang JYH, and Yang P. Ensemble deep learning in bioinformatics. Nat Mach Intell. 2020:**2**(9):500–508. https://doi.org/10.1038/s42256-020-0217-y

Farquhar S, Kossen J, Kuhn L, and Gal Y. Detecting hallucinations in large language models using semantic entropy. Nature. 2024:**630**(8017):625–630. https://doi.org/10.1038/s41586-024-07421-0

Gao Y, Xiong Y, Gao X, Jia K, Pan J, Bi Y, Dai Y, Sun J, Wang M, and Wang H. Retrieval-Augmented Generation for Large Language Models: A Survey. arXiv.org. 2023. https://arxiv.org/abs/2312.10997v5. Retrieved February 11, 2026

Gou Z, Shao Z, Gong Y, Shen Y, Yang Y, Duan N, and Chen W. CRITIC: Large Language Models Can Self-Correct with Tool-Interactive Critiquing. arXiv.org. 2023. https://arxiv.org/abs/2305.11738v4. Retrieved February 11, 2026

Gu J, Jiang X, Shi Z, Tan H, Zhai X, Xu C, Li W, Shen Y, Ma S, Liu H, et al. A Survey on LLM-as-a-Judge. arXiv.org. 2024. https://arxiv.org/abs/2411.15594v6. Retrieved February 11, 2026

Ling Z, Fang Y, Li X, Huang Z, Lee M, Memisevic R, and Su H. Deductive Verification of Chain-of-Thought Reasoning. arXiv.org. 2023. https://arxiv.org/abs/2306.03872v3. Retrieved February 11, 2026

Liu L, Pan Y, Li X, and Chen G. Uncertainty Estimation and Quantification for LLMs: A Simple Supervised Approach. arXiv.org. 2024. https://arxiv.org/abs/2404.15993v4. Retrieved February 11, 2026

Soman K, Rose PW, Morris JH, Akbas RE, Smith B, Peetoom B, Villouta-Reyes C, Cerono G, Shi Y, Rizk-Jackson A, et al. Biomedical knowledge graph-optimized prompt generation for large language models. Bioinformatics. 2024:**40**(9):btae560. https://doi.org/10.1093/bioinformatics/btae560

Trad F and Chehab A. To Ensemble or Not: Assessing Majority Voting Strategies for Phishing Detection with Large Language Models. . In. , pp. 158–173. https://doi.org/10.1007/978-3-031-82150-9_13

Vacareanu R, Pratik A, Spiliopoulou E, Qi Z, Paolini G, John NA, Ma J, Benajiba Y, and Ballesteros M. General Purpose Verification for Chain of Thought Prompting. arXiv.org. 2024. https://arxiv.org/abs/2405.00204v1. Retrieved February 11, 2026

Wang J, Wang J, Athiwaratkun B, Zhang C, and Zou J. Mixture-of-Agents Enhances Large Language Model Capabilities. 2024. https://doi.org/10.48550/arXiv.2406.04692

Wang X, Wei J, Schuurmans D, Le Q, Chi E, Narang S, Chowdhery A, and Zhou D. Self-Consistency Improves Chain of Thought Reasoning in Language Models. 2023. https://doi.org/10.48550/arXiv.2203.11171

Yang D, Tsai Y-HH, and Yamada M. On Verbalized Confidence Scores for LLMs. arXiv.org. 2024. https://arxiv.org/abs/2412.14737v1. Retrieved February 11, 2026

File S6: Public supercomputing infrastructure available for AI research

These facilities enable educators and researchers to apply for free access to supercomputing resources, allowing them to develop AI/ML research pipelines for plant science research. These facilities typically also organize training workshops that eligible researchers can access. Commercial services such as Amazon Web Services (AWS) and Google Cloud may also provide rapid access to supercomputing where institutional facilities are not available.

#	Name	Country/Region	Link
1	Clementina XXI	Argentina	https://www.argentina.gob.ar/ciencia/sistemasnacionales/clementina-xxi-descubri-la-supercomputacion
2	Supercomputador Santos Dumont (SINAPAD)	Brazil	https://sdumont.lncc.br
3	Digital Research Alliance of Canada	Canada	https://www.alliancecan.ca/en
4	SCAI Lab / National Laboratory for High Performance Computing (NHLPC)	Chile	https://www.nlhpc.cl/
5	National Supercomputing Centers (NSCC)	China	Multiple
6	Guane-1 (SC3, Universidad Industrial de Santander)	Colombia	https://www.sc3.uis.edu.co/
7	EuroHPC	European Union	https://www.eurohpc-ju.europa.eu/index_en
8	EuroHPC AI Factories	European Union	https://www.eurohpc-ju.europa.eu/ai-factories_en
9	German Network for Bioinformatics Infrastructure (de.NBI cloud)	Germany	https://www.denbi.de/cloud
10	CGIAR AI4Development (Resource linking hub)	Global	https://www.ai4d.ai/
11	National Supercomputing Mission (NSM)	India	https://nsmindia.in/
12	National AI Portal	India	http://indiaai.in/
13	High Performance Computing Infrastructure (HPCI)	Japan	https://www.hpci-office.jp/en
14	HPC Resource Centers	Latin America	https://scalac.redclara.net/en/hpc/hpc-resource-centers-in-al
15	SCALAC	Latin America and Carribean	https://scalac.redclara.net/en/
16	National Integrated CyberInfrastructure	South Africa	https://nicis.ac.za/

	System (NICIS)		
17	National Supercomputing Center (NSC)	South Korea	https://www.ksc.re.kr/
18	Artemisa	Spain	https://artemisa.ific.uv.es
19	Mare Nostrum	Spain	https://www.bsc.es/marenostrum
20	AI Research Resource (AIRR)	United Kingdom	https://www.gov.uk/government/publications/ai-research-resource
21	Cluster-UY (National Supercomputing Center)	Uruguay	http://www.cluster.uy
22	ACCESS	USA	https://access-ci.org/
23	NSF Leadership-Class Computing Facility (LACC)	USA	https://lccf.tacc.utexas.edu/
